# Plasma TMAO Concentrations and Gut Microbiota Composition in Subjects with and Without Metabolic Syndrome: Results from Pilot Study

**DOI:** 10.3390/metabo15060364

**Published:** 2025-05-30

**Authors:** Mohammed E. Hefni, Cornelia M. Witthöft, Patrik Hellström, Ingegerd Johansson, Anders Esberg

**Affiliations:** 1 Department of Chemistry and Biomedical Sciences, Linnaeus University, 392 31 Kalmar, Sweden; cornelia.witthoft@lnu.se; 2 Food Industries Department, Faculty of Agriculture, Mansoura University, P.O. Box 46, Mansoura 35516, Egypt; 3 Department of Health and Caring Sciences, Linnaeus University, 392 31 Kalmar, Sweden; patrik.hellstrom@lnu.se; 4 Department of Odontology, Umeå University, 901 87 Umeå, Sweden; ingegerd.johansson@umu.se (I.J.); anders.esberg@umu.se (A.E.)

**Keywords:** gut microbiota, metabolic syndrome, TMAO, TMA, choline, carnitine

## Abstract

**Background/Objectives**: Trimethylamine N-oxide (TMAO) is a gut microbiota-dependent metabolite considered as a risk metabolite for various non-communicable diseases. This study aims to identify differences in the gut microbiota composition and concentrations of TMAO and related metabolites in subjects with and without metabolic syndrome (MetS). **Methods**: Plasma samples were collected following an overnight fast on two occasions from subjects with (n = 12) and without (n = 21) MetS. Feces samples were collected on the day before the first blood sampling. The gut microbiota was profiled using 16S rRNA full-gene amplification sequencing. TMAO and related methylamines were quantified using UPLC-MSMS. The fasted plasma glucose, plasma lipid profile, and HbA1c were determined, and blood pressure, circumference, height, and weight were measured. **Results**: A divergent gut microbiota composition was observed in feces samples from both groups. In contrast to subjects without MetS, subjects with MetS had a reduced microbial diversity, with lower *Blautia glucerasea* and higher *Ruminococcus torques*—a pattern associated with (increased) inflammation. Trimethylamine (TMA)-producing bacteria were low in abundance across both groups. While plasma TMAO and related methylamines displayed no significant differences between both groups, L-carnitine was elevated (*p* = 0.0191) in subjects with MetS. A strong positive correlation was detected between TMAO and TMA (*r* = 0.439, *p* = 0.003), with a tendency to correlate with carnitine (*r* = 0.212, *p* = 0.087). **Conclusions**: Subjects with MetS were characterized by gut microbiota favoring inflammation-associated species but not TMA producers. This suggests that TMAO may not play a role in MetS subjects without overt comorbidities, e.g., CVD or T2D. The influence of the gut microbiota on early MetS is likely mediated through inflammatory mechanisms driven by specific bacterial shifts rather than TMAO production.

## 1. Introduction

Metabolic syndrome (MetS) is a cluster of conditions characterized by an abnormal lipid profile, elevated blood glucose, blood pressure, and anthropometric measures which collectively increase the risk of cardiovascular diseases (CVDs) and T2D [1]. According to the National Cholesterol Education Program (NCEP) Adult Treatment Panel III (ATP III), a minimum of three of the following criteria should be met for a diagnosis of MetS: waist circumference > 102 cm in men and >88 cm in women; fasting plasma glucose ≥ 5.6 mmol/L; triglycerides > 1.7 mmol/L or medication; HDL cholesterol < 1.03 mmol/L in men and <1.29 mmol/L in women or medication; systolic/diastolic pressure ≥ 130/85 mm Hg or medication [2].

The gut microbiota is becoming increasingly recognized as a crucial metabolic regulator, involved in the development and progression of MetS and various chronic diseases, such as CVD and T2D [3,4,5,6,7,8]. Individuals with MetS are reported to have an altered gut microbiota profile which leads to a metabolic imbalance (in the host) [4,7]. For example, the *Firmicutes*/*Bacteroidetes* ratio has been reported to be higher in subjects with MetS compared to those without [9]. Other studies found *Actinobacteria*, *Bifidobacteriales*, *Bifidobacteriaceae*, *Desulfovibrio*, and *Ruminococcaceae UCG010* to be associated with an increased risk of MetS [10]; however, results are inconsistent.

TMAO is a key metabolite produced by the gut microbiota and has gained attention as a risk marker for adverse health outcomes, including CVD and T2D [5,6,11]. Increased fasting plasma TMAO concentrations not only predict disease risk; they are also involved in the pathophysiology through the promotion of vascular inflammation, platelet hyperactivity, abnormal cholesterol metabolism, and atherosclerosis [5,12,13,14]. TMAO can be obtained directly from foods, such as seafood, or be synthesized through the oxidation of trimethylamine (TMA) [15,16]. TMA is produced by the gut microbiota from nutrients, e.g., choline, phosphatidylcholine, and carnitine [17], which are abundant in animal-based foods [18]. Two primary pathways for TMA synthesis have been described. In the first, choline is used as a substrate involving the glycyl radical enzyme choline TMA-lyase (CutC) and its activator CutD [19]. In the second, carnitine is used as a substrate for two-component Rieske-type oxygenase/reductase (CntA/B) [20]. Additionally, a third pathway has recently been proposed via betaine as a substrate involving the enzyme complex YeaW/X with a close sequence similarity to carnitine oxygenase (CntA/B) [20]. The choline TMA-lyase cluster *cutC/D* is widely distributed across bacterial taxa, such as *Anaerococcus hydrogenalis*, *Clostridium asparagiformis*, *Clostridium hathewayi*, *Clostridium sporogenes*, *Desulfovibrio desulfuricans*, *Escherichia fergusoni*, *Klebsiella pneumoniae*, and *Proteus penneri* [17,21]. Additionally, CntA/B is present in *Burkholderia* spp., *Cupriavidus* spp., *Pseudomonas* spp., *Stenotrophomonas* spp., *Yersinia* spp., *Yokenella* spp., *Acinetobacter baumannii*, *Klebsiella pneumoniae*, *Shigella* spp., and *Sporosarcina* spp. [18]. Hence, the gut microbiota can significantly influence the amount of produced TMA and, consequently, the levels of TMAO in the plasma. Accordingly, subjects with different gut microbiota profiles may have varying TMAO levels, which possibly reflect their underlying metabolic health.

Most studies have focused on the relationship between TMAO and CVD, T2D, or other advanced cardiac events, while intermediary factors such as obesity, the Body Mass Index (BMI), and the lipid profile were examined as secondary or even tertiary outcomes [22]. However, recent studies have reported positive correlations between plasma TMAO levels and the BMI [23,24] and lipid levels [25]. In this study, we focus on MetS subjects with no evidence of CVD and T2D. This will reduce the confounding effects of advanced comorbidities that can independently alter circulating methylamines as well as the gut microbiota composition [23,26,27]. This study aims to identify differences in gut microbiota compositions and concentrations of TMAO and related metabolites in subjects with and without MetS.

## 2. Materials and Methods

### 2.1. Subjects

**Figure 1:**
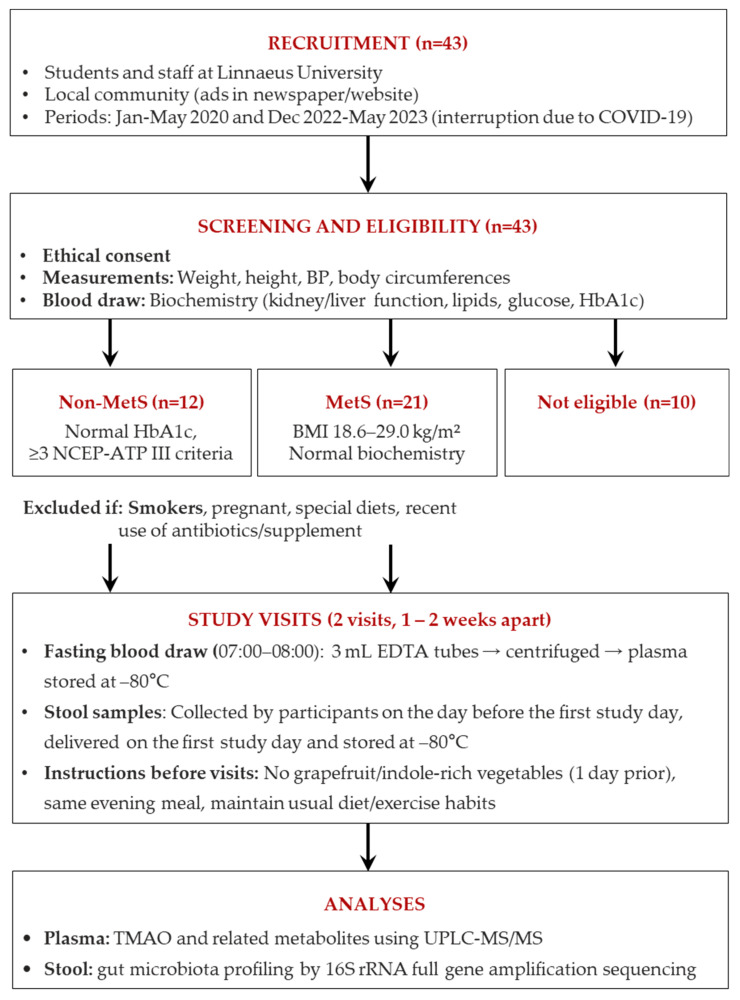
A flow chart of the study outline. Bp: blood pressure, ATP III: Adult Treatment Panel III, and TMAO: trimethylamine N-oxide.

Thirty-three subjects aged 18–75 years with (MetS, n = 12) or without (non-MetS, n = 21) MetS were recruited from the student and staff population at Linnaeus University, Kalmar, Sweden, and the surrounding communities through advertisements in the local newspaper and the university website. Recruitment was performed from January to May 2020 and December 2022 to May 2023 with the interruption caused by the coronavirus pandemic. Subjects were not eligible for the study if they participated in another study; were smokers, pregnant, breastfeeding, or planning a pregnancy; took antibiotics or probiotics two months before starting the study or nutritional supplements two weeks before starting the study; or followed a special diet, e.g., vegan, vegetarian, or weight loss. If consenting, eligible participants without MetS should have a BMI between 18.6 and 29.0 kg/m^2^ and selected biochemical measures within reference range, including kidney function (age-related P-creatinine values), liver enzymes (aspartate transaminase, alanine transaminase, and γ-glutamyl transferase activity), blood pressure, blood status, plasma lipids, and plasma glucose and HbA1c within age-related reference range (to exclude diabetes). MetS subjects were required to meet age-related P-creatinine values for normal kidney function, HbA1c within age-related reference range to exclude diabetes, and a minimum of three of the NCEP-ATP III criteria, i.e., a waist circumference > 102 cm in men or >88 cm in women; fasting P-glucose ≥ 5.6 mmol/L; P-triglycerides > 1.7 mmol/L or medication; P-HDL cholesterol < 1.03 mmol/L in men or <1.29 mmol/L in women or medication; or blood pressure ≥ 130/85 mm Hg or medication (Figure 1).

The study was approved by the Swedish Ethical Review Authority, Sweden (Dnr: 2019-04354). A signed informed consent form was obtained from all subjects. This trial was registered at ClinicalTrials.gov as NCT06660251.

### 2.2. Study Visits and Blood and Feces Sample Collection

At screening, weight, height, body circumference, and blood pressure were measured, and blood was drawn for biochemical measures.

After the screening, subjects came to the health clinic for two blood sampling occasions one to two weeks apart. Blood was drawn between 07.00 and 08.00 a.m. following an overnight fasting. Participants were instructed to avoid grapefruit juice and indole-containing vegetables (i.e., broccoli, Brussels sprouts, cabbage, and cauliflower) the day before sampling as these foods can decrease flavin-containing monooxygenase 3 (FMO3) enzyme activity and alter TMAO metabolism [28]. They were also instructed to eat the same evening meal the day before blood sampling. Aside from this, subjects were asked to maintain their normal diet and exercise habits throughout the study period. During the visits, 3 mL blood samples were drawn into EDTA vacutainer tubes for analyses of TMAO and related metabolites. After collection, blood was immediately centrifuged at 2000× *g* for 10 min, and plasma was aliquoted into 1.5 mL Eppendorf tubes and stored at −80 °C until analysis.

Subjects were provided with a stool collection kit (DNA/RNA shield-fecal collection tube, BioSite-R110, Nordic Biosite, Täby, Sweden) at screening, along with instructions for feces collection and a thermal insulation bag for transportation. They were asked to collect a stool sample on the day before the first study visit, or as close as possible, and store the sample at −20 °C until it was transferred to the clinic. Once delivered, the samples were transferred and stored at −80 °C until analysis.

### 2.3. Clinical Analyses

#### 2.3.1. Blood Chemistry

The analyses of fasted blood samples for liver enzymes, lipid profile, glucose, and HbA1c) were carried out using routine procedures at the Department of Clinical Chemistry and Transfusion Medicine, Diagnostic Center, County Hospital in Kalmar. Blood status was analyzed using the Swelab Alfa cell counting process (Boule Diagnostics, Spånga, Sweden) at the Biochemistry Laboratory at Linnaeus University, Kalmar.

#### 2.3.2. Quantification of TMAO and Related Metabolites

Analyses of TMAO, TMA, betaine, choline, L-carnitine, acetyl-L-carnitine, and creatinine in plasma samples were performed in duplicate according to previously described methodology [29]. In brief, 25 µL of plasma was homogenized with 100% methanol (100 µL) and 10 µL of internal standard solution (containing 10 µmol/L of each deuterated compound TMA-d9, TMAO-d9, betaine-d11, L-carnitine-d3, acetyl-L-carnitine-d3, and L-carnitine chloride-d9). The samples were vortexed and shaken for 10 min prior to centrifugation (8 min, 13,000× *g*). A total of 100 µL of the supernatant was transferred to a microcentrifuge tube, and 800 μL of acetonitrile, 5 µL of concentrated NH_4_OH, and 5 µL of Iodoacetonitrile was added. The samples were then vortexed and shaken (10 min at room temperature). The reaction was halted by adding 2 µL of formic acid. The samples were centrifuged (5 min, 13,000× *g*), and aliquots of supernatant were transferred to HPLC vials.

Analyses were carried out using an UPLC 1260 II Infinity system (Agilent 1200, Agilent Technologies, Santa Clara, CA, USA) coupled to a triple quadrupole mass spectrometer (Agilent G6495C) equipped with an AJS-ES source (Agilent Jet Stream ionization source), which was operated in Multiple Reaction Monitoring (MRM) and positive ionization mode. Methylamines were separated on a neutral UPLC-HILIC column (ACE, 75 mm × 2.1 mm; particle size 1.7 μm) with a guard column (ACE, 3 mm × 2.1 mm; particle size 1.7 µm). Separation was performed with an isocratic mobile phase containing 70% ammonium formate (10 mmol/L) in MQ water (solvent A) and 30% acetonitrile (solvent B). The column was thermostatically controlled at 25 °C. The flow rate was set at 0.2 mL/min, the injection volume was 1 µL, and the total run time was 6 min.

#### 2.3.3. Microbiota Analysis

##### Feces DNA Extraction

DNA was extracted from 250 mg of feces using the DNeasy PowerSoil Pro Kit (QIAGEN, Kista, Sweden) with 10 min maximum speed vortexing in a Vortex Adapter (QIAGEN). The DNA quality was estimated using a NanoDrop 1000 Spectrophotometer (Thermo Fisher Scientific, Uppsala, Sweden) and the quantity by the Qubit 4 Fluorometer (Invitrogen, Thermo Fisher Scientific, Waltham, MA, USA). DNA in the commercial mock community (ZymoBIOMICS Microbial Community DNA Standard, D6305, Nordic Biosite, Stockholm, Sweden) was used as a positive control while ultrapure water was used as a negative.

##### Bacteria 16S rRNA Full-Gene Amplicon Sequencing

Full 16S rRNA gene sequencing was completed using the Oxford Nanopore Technology as previously described by [30]. Briefly, the v1 through v9 variable regions of 16S rDNA were amplified using 50 ng DNA and KAPA 2x HiFi ready mix (KAPA HiFi HotStart ReadyMix (2X), New England Biolabs, Ipswich, MA, USA) on a MiniAmp™ Thermal Cycler (Thermo Fisher Scientific, Uppsala, Sweden). After gel confirmation of an expected 1465 bp product, amplicons were purified, washed, and eluted in EB buffer (Fisher scientific, Göteborg, Sweden) and quantified using the Qubit dsDNA HS Assay Kit and Qubit 4.0 Fluorometer (Thermo Fisher Scientific, Waltham, MA, USA). Library preparations were performed by barcoding amplicons using the Native Barcoding Kit 96 V14 (SQK-NBD114.96 kit, Oxford Nanopore Technologies, Oxford, UK). Sequencing was performed by loading 100 ng library on a R10.4.1 flow cell (Oxford Nanopore Technologies) on a GridION nanopore sequencer (Oxford Nanopore Technologies) for 72 hrs. Base-calling of nanopore signals and demultiplexing was conducted on the GriION using the MinKNOW (Oxford Nanopore Technologies) Dorado base caller super accurate model and Porechop (version 0.2.4, https://github.com/rrwick/porechop, accessed on 1 February 2025), generating demultiplexed FastQ files, with a quality score ≥ 10 and a read length between 1350 and 1800 bp.

##### Taxonomic Annotation

The Emu pipeline [31] was applied using an open-source software package (https://github.com/treangenlab/emu, accessed on 1 February 2025). Retained sequences were classified against the RDP v11.5 database [32] with NCBI taxonomy [33] pre-built for Emu v3.0+. Species present in a single subject were excluded, and species with <10 reads in a subject were set to 0 to cope with sequence error and mapping accuracy. The MicrobiotaProcess package in R-studio was utilized for read rarefication to a sequencing depth of 28,848 reads, calculation of α-diversity indexes including species richness and Shannon index, and β-diversity (Bray–Curtis distance matrix).

### 2.4. Statistical Analyses

Data are presented as the mean with standard deviation (SD). Group mean differences between MetS and non-MetS for characteristics, including P-lipids, P-glucose, HbA1c, and BMI, were tested with an unpaired *t*-test. A linear mixed-effects model was applied to determine differences in TMAO and related metabolites between both groups, with age and sex as covariates. Associations between TMAO, choline, carnitine, and TMA and MetS characteristics were assessed using partial Spearman’s rank correlation analysis with age, sex, and day of sampling as covariates. For gut microbiota, Orthogonal Partial Least Squares Discriminant Analysis (OPLS) regression and Bray–Curtis dissimilarity index comparisons were run for microbiota comparisons and identification of the differing taxa between the two groups. The MaAsLin3 (Microbiome Multivariable Associations with Linear Models) [34] in R studio [35] was used to identify microbial taxa associated with having a MetS and non-MetS status in multivariable generalized linear model regression including age, sex, and total number of reads. Data transformations and nonparametric tests were employed when necessary to meet analysis assumptions. The analyses were performed using R (RStudio 2024.04.2-764), and *p*-values < 0.05 were considered to indicate a statistically significant difference.

## 3. Results

### 3.1. Clinical Parameters

**Figure 2:**
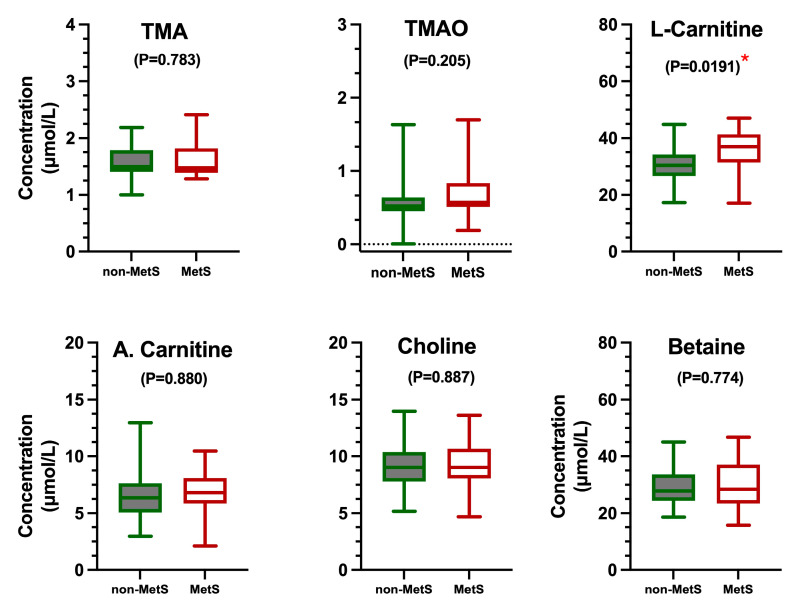
Methylamine concentrations of subjects with MetS (n = 12) and without MetS (n = 21). Data are derived from fasting plasma samples (analyzed in duplicate) collected at two occasions. A linear mixed-effects model was applied to determine the significant differences in TMAO and related metabolites between the MetS and non-MetS group with age and sex included as covariates. No significant differences were observed between both groups, except for L-carnitine (* *p* = 0.0191). A. Carnitine: acetyl-L-carnitine.

The clinical parameters defining MetS were confirmed to differ between subjects with and without MetS, whereas age and height were similar in both groups (Table 1). Thus, subjects with MetS displayed a significantly higher waist circumference, weight, BMI, fasting plasma glucose, plasma triglycerides, and HbA1c alongside significantly lower plasma HDL-cholesterol levels compared to subjects without MetS. Moreover, the MetS group had a higher plasma concentration of L-carnitine than the non-MetS group (*p* = 0.019) (Figure 2), whereas TMAO, TMA, choline, betaine, and acetyl-L-carnitine concentrations did not differ between the two groups (Figure 2). Additionally, correlation analyses between MetS features and TMAO, TMA, choline, and L-carnitine, after adjusting for age, sex, and inter-day variation, revealed significant correlations. These were between TMAO with weight and the BMI and L-carnitine with the waist circumference and weight, glucose, HbA1c, HDL cholesterol, and triglycerides (Table 2). Furthermore, TMAO correlated with TMA and showed a tendency to correlate with carnitine (Table 3).

### 3.2. Gut Microbiota

#### 3.2.1. Gut Microbiota Composition

**Figure 3:**
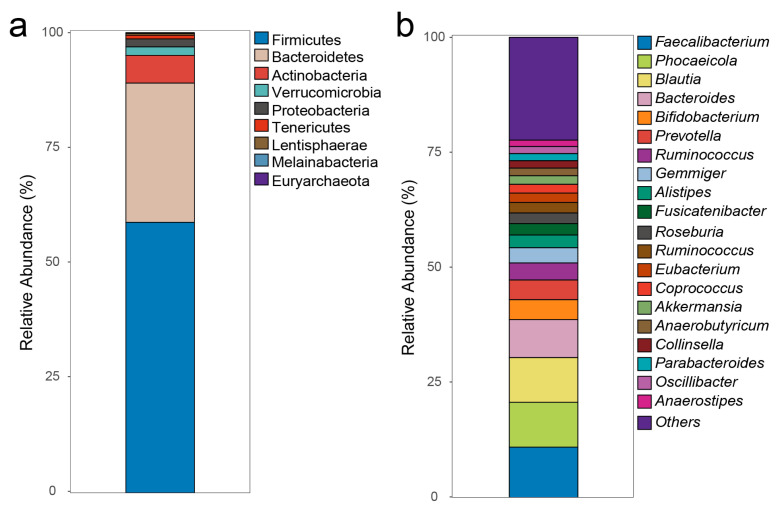
The gut microbiota composition. After rarefying to the same sequencing depth, the relative abundance of the overall composition at the phylum (**a**) and genus level (**b**) is shown in stacked bar plots.

Nanopore sequencing generated 3,493,134 quality-filtered EMU mapped reads, producing a median (min, max) of 79,244 (29,943, 327,096) reads per sample. After rarefying the data to a consistent sampling depth of 28,900 reads per sample, the reads represented nine phyla, in which Firmicutes, Bacteroidetes, and Actinobacteria covered 95.1% of the total reads (Figure 3a). The mapped reads represented 121 genera (top 20 relative abundant genera are presented in Figure 3b), 14 of which were present in >95% of the samples and covered 64% of the total number of reads (prevalence, %-reads): *Faecalibacterium* (100%, 10.9%), *Phocaeicola* (100%, 9.9%), *Blautia* (100%, 9.7%), *Bacteroides* (100%, 8.3%), *Anaerobutyricum* (100%, 1.7%), *Anaerostipes* (100%, 1.4%), *Fusicatenibacter* (100%, 2.5%), *Ruminococcus* (100%, 6.0%), *Alistipes* (97%, 2.7%), *Coprococcus* (97%, 1.9%), *Dorea* (97%, 0.9%), *Eubacterium* (97%, 2.5%), *Gemmiger* (97%, 3.3%), and *Roseburia* (97%, 2.3%). The 122 genera encompassed 232 species of which *Anaerobutyricum hallii*, *Anaerostipes hadrus*, *Blautia luti*, *Blautia* sp. *SC05B48*, *Blautia wexlerae*, *Faecalibacterium prausnitzii*, *Fusicatenibacter saccharivorans*, *Phocaeicola dorei*, *Dorea longicatena*, and *Gemmiger formicilis* were present in >95% of the subjects (Table S1).

#### 3.2.2. Gut Microbiota Composition of Non-MetS and MetS Subjects

**Figure 4:**
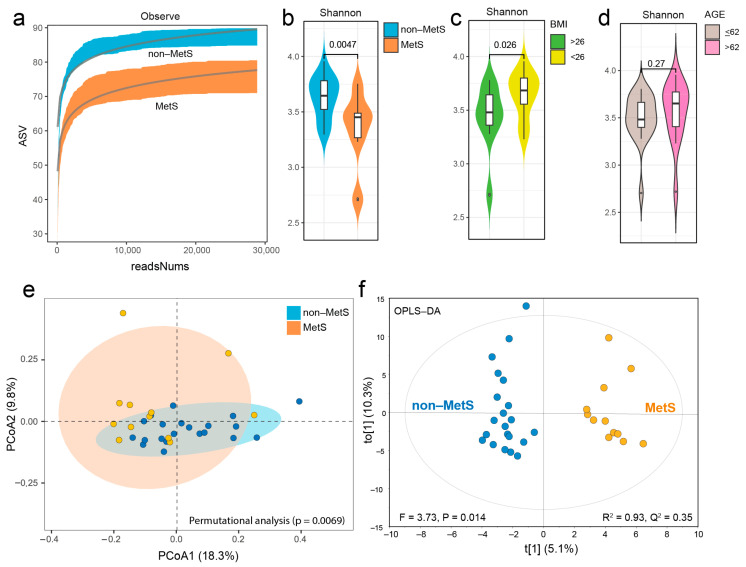
The gut microbiota composition. Rarefaction curves depicting the observed number of species (**a**) and the Shannon diversity index (**b**) for subjects with non-MetS and MetS. The panel (**c**) shows the Shannon diversity index across subgroups based on the Body Mass Index (BMI), while panel (**d**) illustrates variations by age. Additionally, the comparison of the gut microbiota composition is represented using the Bray–Curtis dissimilarity index, accounting for 18.3% and 9.8% of the variation observed (*p* = 0.0069) (**e**). The panel (**f**) displays a score plot from the OPLS-DA model, demonstrating a significant separation between non-MetS and MetS participants (R^2^ = 0.93, Q^2^ = 0.35, *p* = 0.014).

After rarefying the data to a consistent sampling depth of 28,900 reads per sample, differences were observed between non-MetS and MetS subjects regarding both the number of species and the Shannon diversity index. Specifically, the MetS group exhibited a reduced number of species (*p* = 0.041) and a lower Shannon diversity index (*p* = 0.0047) (Figure 4a,b). Additionally, we verified a previously established association between an increased BMI and reduced Shannon diversity (Figure 4c), whereas age and sex were not significantly associated with diversity (Figure 4d and Figure S1). To evaluate potential compositional shifts in the gut microbiota by the MetS status, the Bray–Curtis dissimilarity index was estimated, revealing a significant difference between the MetS and non-MetS groups (*p* = 0.007) (Figure 4e). To further evaluate potential differences, an OPLS-DA analysis was performed, yielding an R^2^ of 93% and a cross-validated Q^2^ of 35%, supporting a significant compositional shift between MetS and non-MetS subjects (Figure 4f).

#### 3.2.3. Taxonomic Differences Between Non-MetS and MetS Subjects

To follow up on the compositional shift indicated by the Bray–Curtis and OPLS-DA analysis, the pheatmap (R package) and Maaslin3 pipeline were applied to identify potential patterns and differences when adjusting for sex, age, and the total number of reads. The heatmap illustrates the associations between microbial species and TMAO and related metabolites. Hierarchical clustering was applied to both rows and columns to group species and clinical markers with similar association profiles (Figure S2). Maaslin3 identified 15 species that differentiated the non-MetS and MetS subjects based on their abundance or prevalence (no FDRs were applied due to the limited number of subjects, *p* < 0.05). Three species displayed a positive association with MetS subjects: *Bifidobacterium ruminantium*, *Bacteroides fragilis*, and *Ruminococcus torques*. In contrast, 12 species were associated with non-MetS subjects: 3 *Blautia* (*stercoris*, *glucerasea*, and *producta*), *Ruminococcus champanellensis* and *bicirculans*, *Roseburia intestinalis*, *Coprococcus eutactus*, *Lachnospiraceae bacterium* GAM79, *Christensenella* sp. *Marseille* P3954, *Monoglobus pectinilyticus*, *Desulfovibrio piger*, and *Kiloniella majae*. The strongest species differentiation between non-MetS and MetS subjects were *Ruminococcus torques*, which showed a 3.8-fold elevated level in MetS (*p* = 0.004) and the *Blautia glucerasea* which showed an increased prevalence in the non-MetS group with an odds ratio (OR) (95% CI) of 15.1 (2.3–100.2) (*p* = 0.007) (Table S2).

## 4. Discussion

This study aimed to identify differences in the gut microbiota composition and concentrations of TMAO and related metabolites in subjects with and without metabolic syndrome. MetS is a condition characterized by an elevated BMI, waist circumference, blood glucose, blood pressure, triglycerides, and reduced HDL cholesterol. By focusing on MetS subjects without any comorbidities, e.g., diabetes, CVD, or kidney dysfunction, we isolated microbial and metabolic dysregulation to MetS alone. Furthermore, by excluding potential subjects with other diseases, the confounding effects of advanced comorbidities that can independently alter both circulating methylamines and gut microbiota compositions were avoided [23,26,27].

Increased circulating L-carnitine levels were observed among subjects with MetS. This elevation was positively correlated with key MetS features, including adiposity, dyslipidemia, and elevated glycemic indices. L-carnitine, a metabolite critical for fatty acid metabolism, is widely utilized as an ergogenic aid and in weight loss interventions [36]. However, our study, along with others [37,38], revealed unfavorable associations between elevated plasma L-carnitine and MetS. L-carnitine is one of the two main substrates used for the formation of TMAO. Upon the ingestion of L-carnitine-rich foods, e.g., red meat, gut bacteria via the CntA/B cleaves L-carnitine into TMA, which is subsequently oxidized to TMAO by hepatic FMO3 [13]. Despite this pathway, confirmed findings by others [37] have demonstrated no significant correlation between L-carnitine and TMAO in subjects with MetS [37], suggesting that L-carnitine may exert metabolic effects independently of TMAO [37,38]. A recent study [39] in individuals with precursor cardiometabolic conditions (e.g., obesity, MetS) found no significant association between circulating TMAO levels and disease risk, despite the elevated carnitine in MetS patients. This supports our finding that the metabolic effects of L-carnitine are not mechanistically tied to TMAO in early disease stages. Despite the increase in L-carnitine—the primary substrate for TMAO formation—the plasma TMAO concentration did not increase. L-carnitine is an animal-based nutrient; therefore, the subjects’ diets may also play a role. However, because we did not determine the dietary intake of animal-based foods in this study we are unable to assess contributions from exogenous L-carnitine. To mitigate potential confounders, participants were instructed to avoid consuming grapefruit juice and indole-rich vegetables (e.g., broccoli, Brussels sprouts, cabbage, and cauliflower), which can inhibit FMO3 activity. They were also asked to consume a similar evening meal prior to sampling. Additionally, the collection of fasting blood samples at two occasions (adjusted for inter-day variation) enhanced the reliability of our measurements. Moreover, the endogenous L-carnitine synthesis may contribute to elevated levels of L-carnitine. Reddy et al. observed reduced plasma lysine and methionine (precursors for L-carnitine biosynthesis) in MetS subjects free of comorbidities inversely correlating with L-carnitine levels (lysine: *r* = −0.47, *p* = 0.0006; methionine: *r* = −0.37, *p* = 0.0075), suggesting an increased utilization of these amino acids for the de novo synthesis of L-carnitine [40].

A significant correlation was also observed between TMAO levels and the BMI and weight, thereby verifying previous findings [22,23,26]. However, we did not find any association between TMAO and glycemic indices (fasting glucose and HbA1c) or plasma lipids, which also confirms previous findings [37]. This contrasts with research in advanced MetS cohorts with comorbid diabetes, where TMAO has been linked to metabolic dysregulation [23,26,27]. This discrepancy is reflected by our focus on MetS subjects, excluding confounders such as diabetes and CVDs, which are known to affect TMAO levels [23,26,27]. The lack of an association between TMAO and glucose or lipid metabolism suggests that TMAO is possibly stage-dependent during MetS progression or serves as a biomarker rather than a causal driver of metabolic dysregulation [37]. Importantly, the current results were adjusted for age, sex, and the between-day variation—by comparing residuals after removing these effects—thus strengthening the validity of these associations despite our relatively small sample size. Furthermore, our data revealed a significant correlation between TMA and TMAO levels, highlighting the critical role that the gut–liver axis plays in metabolic regulation.

Although there was a relatively small sample size, we observed a reduction in microbial diversity and a shift in the gut microbiota composition between non-MetS and MetS subjects, which is consistent with previous findings [4,7,9,10]. A key finding regarding the gut microbiota compositional shift was linked to the increased health-promoting *Blautia* species (*glucerasea*, *stercoris*, and *producta*) [41] in non-MetS participants. The *Blautia* genus, a member of the Firmicutes phylum, has been linked to metabolic health [41]. *Blautia* species can ferment dietary fibers to produce short-chain fatty acids (SCFAs), such as acetate, propionate, and butyrate [41], which are reported to have beneficial effects on the metabolism by reducing systemic inflammation, regulating appetite, and improving insulin sensitivity [42]. Studies show that metabolically healthy subjects typically have higher abundances of *Blautia* species, while those with metabolic disorders often exhibit a decreased diversity in gut bacteria [43]. The ability of *Blautia* to produce beneficial SCFAs and its correlation with metabolic health markers suggest that a balanced gut microbiota, including *Blautia*, may help prevent the development of MetS.

On the other hand, *Ruminococcus torques*, a mucin-degrading bacterium, was found to be significantly higher in MetS participants. *R. torques* is shown to be efficient in the degradation of human colonic MUC2 [44], a key component of the protective intestinal mucus layer. Consequently, *R. torques* has been linked to a decreased mucus thickness and increased mucus penetrability, resulting in inflammation and subsequent disease development [44,45]. Indeed, *R. torques* is implicated in inflammatory bowel diseases, Crohn’s disease, the degradation of the blood group antigen components (A and H) in intestinal glycosphingolipids, and hemodialysis-related dysbiosis but is positively associated with TMAO levels [46,47]. In a recent study investigating children with metabolic dysfunction-associated steatotic liver disease (MASLD) [48], *R. torques* was significantly higher in those with MALSD and was positively associated with increased levels of deoxycholic acid (DCA). DCA has been linked to several harmful effects at the cellular level, including inflammation and immune dysregulation and risk factors for dyslipidemia and reduced insulin sensitivity [49]. Although the present study did not analyze any inflammatory markers, a previous study has demonstrated elevated endotoxin levels and a Toll-like receptor 4 activity in subjects with MetS compared to those without, indicating increased inflammation and gut permeability [37]. The authors [37] did not determine the gut microbiota composition, which was a key focus in our current investigation. Hence, our findings may contribute to understanding the relationships between MetS, blood metabolites, and the gut microbiota and align with the existing literature exploring the interplay between the gut microbiota, host metabolism, and cardiometabolic risk. For instance, Org et al. [50] identified significant associations between the gut microbial composition, plasma metabolites—including TMAO precursors—and metabolic syndrome traits in the METSIM cohort, highlighting the complexity of microbiota–metabolite–host interactions. Similarly, Li et al. [51] demonstrated that diet–microbiome interactions influence circulating TMAO levels in a longitudinal human cohort, highlighting the important role of the gut microbiome in mediating the health effects of diets. Throughout the recruitment process, we implemented stringent eligibility criteria and thoroughly excluded confounding factors, such as recent antibiotic use, smoking, and dietary restrictions. This rigor ultimately enhanced the validity of our findings. Moreover, our multi-faceted approach, which combines a blood metabolite analysis (including TMAO, choline, and carnitine) with gut microbiota sequencing utilizing advanced Oxford Nanopore Technology, allows for a comprehensive exploration of the metabolomic landscape and its microbial correlates. However, several limitations must be acknowledged. The relatively small sample size of 33 subjects may limit the generalizability of our findings and the statistical power to detect subtle associations. Additionally, the cross-sectional nature of this study restricts our ability to draw causal inferences regarding the relationships between the gut microbiota composition, metabolite levels, and MetS characteristics. Longitudinal studies are necessary to elucidate the temporal dynamics of these associations. To summarize, while this study provides valuable insights into the interplay between MetS, the gut microbiota, and plasma metabolites, future research involving larger sample sizes and longitudinal designs is crucial to validate and further expand upon these findings. Overall, the results should be viewed with an appreciation of their certain limitations and strengths.

## 5. Conclusions

A shift in the gut microbiota composition characterized by less diversity and reduced levels of beneficial *Blautia* spp. alongside an increased abundance of inflammation-associated *R. torques* was observed among subjects with MetS. The circulating TMAO did not significantly differ between subjects with and without MetS. While TMAO is linked to cardiovascular disease and insulin resistance, its absence in MetS subjects free of comorbidities may suggest that it is not a direct driver of MetS but rather a secondary marker. This finding suggests that the effect of the gut microbiota on MetS occurs through inflammatory mechanisms driven by a specific bacterial shift, as opposed to TMAO production. Furthermore, TMAO levels may only rise after MetS has progressed to advanced stages, such as T2D or CVD.

## Figures and Tables

**Table 1:** Characteristics of subjects.

Variable	Non-MetS (n = 21)	MetS (n = 12)	Unpaired *T* Test
Age (years) #	57 (13.2)	63 (8.7)	0.1772 ^ns^
Waist circumference (cm)	87 (9.5)	111 (12.5)	<0.0001 ***
Weight (kg)	73 (10.9)	95 (18.7)	0.0002 ***
Height (m)	1.71 (0.07)	1.67 (0.11)	0.2401 ^ns^
BMI (kg/m^2^)	24.8 (3.2)	33.5 (5.1)	<0.0001 ***
P-glucose (mmol/L)	5.3 (0.38)	6.0 (0.45)	<0.0001 ***
HbA1c (mmol/L) #	35.1 (2.2)	39.1 (5.3)	0.0045 **
P-cholesterol (mmol/L)	5.8 (1.12)	5.1 (1.04)	0.0934 ^ns^
P-HDL-cholesterol (mmol/L)	1.9 (0.47)	1.3 (0.42)	0.0013 **
P-triglycerides (mmol/L) #	0.98 (0.49)	1.91 (0.73)	0.0001 ***

Data are presented as mean (standard deviation). # for log transformed values. ^ns^ *p* > 0.05, ** *p* ≤ 0.01, and *** *p* ≤ 0.001.

**Table 2:** Spearman correlation coefficients (rho) and associated *p*-values between subject characteristics at screening and plasma concentrations of TMAO, L-carnitine, choline, and TMA.

Variable	TMAO		Carnitine		Choline		TMA	
	Rho	*p*-Value	Rho	*p*-Value	Rho	*p*-Value	Rho	*p*-Value
Waist circumference	0.18	0.15 ^ns^	0.61	<0.0001 ***	0.13	0.31 ^ns^	−0.05	0.67 ^ns^
Weight	0.25	0.05 *	0.60	<0.0001 ***	0.21	0.09 ^ns^	−0.08	0.53 ^ns^
BMI	0.25	0.04 *	0.59	<0.0001 ***	0.16	0.20 ^ns^	−0.06	0.64 ^ns^
P-glucose	0.11	0.37 ^ns^	0.37	<0.0001 ***	0.00	0.99 ^ns^	−0.20	0.11 ^ns^
HbA1c	0.19	0.13 ^ns^	0.37	<0.0001 ***	−0.08	0.51 ^ns^	0.29	0.02 *
P-cholesterol	−0.11	0.38 ^ns^	−0.17	0.17 ^ns^	0.16	0.19 ^ns^	−0.14	0.28 ^ns^
P-HDL cholesterol	−0.06	0.63 ^ns^	−0.42	<0.0001 ***	−0.07	0.57 ^ns^	0.13	0.29 ^ns^
P-triglycerides	0.14	0.25 ^ns^	0.55	<0.0001 ***	0.10	0.41 ^ns^	−0.01	0.96 ^ns^
Systolic blood pressure	0.05	0.68 ^ns^	0.15	0.22 ^ns^	0.06	0.63 ^ns^	0.03	0.84 ^ns^
Diastolic blood pressure	−0.10	0.43 ^ns^	0.11	0.39 ^ns^	0.26	0.04 *	−0.17	0.18 ^ns^

^ns^ *p* > 0.05, * *p* ≤ 0.05, and *** *p* ≤ 0.001.

**Table 3:** Pairwise partial Spearman correlation coefficients (rho) and associated *p*-values between TMAO and related precursors.

Variables		Adjusted for Age, Sex, Sampling Day		Not Adjusted for Age, Sex, Sampling Day	
		Rho	*p*-Value	Rho	*p*-Value
TMAO	Carnitine	0.212	0.087	0.237	0.056
TMAO	Choline	0.163	0.192	0.300	0.014
TMAO	TMA	0.439	<0.001	0.332	0.006
Carnitine	Choline	0.039	0.756	0.082	0.514
Carnitine	TMA	0.063	0.617	0.036	0.775
Choline	TMA	0.137	0.272	0.128	0.307

## Data Availability

The original contributions presented in this study are included in the article and Supplementary Materials. Further inquiries can be directed to the corresponding author.

## Supplementary Materials

The following supporting information can be downloaded at https://www.mdpi.com/article/10.3390/metabo15060364/s1, Figure S1: Raincloud plots showing alpha diversity (Chao1, ACE, Shannon indexes) by sex. Each plot combines a half-violin (distribution), a boxplot (median and interquartile range), and individual data points. The plots display microbial diversity in feces samples from women and men; Figure S2: Heatmap illustrating the associations between microbial species and TMAO and related compounds; Table S1: Sequencing characteristics. Nanopore sequencing of 33 samples to generate 3,493,134 quality-filtered EMU mapped reads. After rarefying the data to a consistent sampling depth of 28,900 reads per sample, the reads represented 9 phyla, 121 genera, and 232 species. The rarified number of sequences (n) and the proportion (%) of the total number of reads, prevalence (n), and proportion (%) of participants are presented. Rarefying was performed using the MicrobiotaProcess R package; Table S2: Maaslin3 (Microbiome Multivariable Associations with Linear Models, R package) results determine multivariable associations between metabolic and non-metabolic syndromes and the abundance and prevalence of species while accounting for covariates such as the number of reads, age, and sex.

## Abbreviations

The following abbreviations are used in this manuscript:

ATP III	Adult Treatment Panel III
CntA/B	A two-component Rieske-type oxygenase/reductase system converts carnitine to TMA
CutC	Glycyl radical enzyme converts choline to TMA (choline TMA-lyase)
CutD	CutC-activating protein (the activator protein for choline TMA-lyase)
YeaW/X	A two-component Rieske-type oxygenase/reductase system converts betaine to TMA
CVD	Cardiovascular disease
FMO3	Flavin-containing monooxygenase 3
MetS	Metabolic syndrome
MUC2	Mucin 2, oligomeric mucus/gel-forming
NCEP	National Cholesterol Education Program
OPLS-DA	Orthogonal Partial Least Squares Discriminant Analysis
PLS	Partial Least Squares Discriminant Analysis
rho	Spearman correlation coefficients
SCFAs	Short-chain fatty acids
SD	Standard deviation
T2D	Type 2 diabetes
TMAO	Trimethylamine N-oxide
TMA	Trimethylamine
