# Supplementary material for: Plasma TMAO Concentrations and Gut Microbiota Composition in Subjects with and Without Metabolic Syndrome: Results from Pilot Study
Source: Metabolites. 2025 May 30;15(6):364. doi: 10.3390/metabo15060364 (PMC12195445; doi:10.3390/metabo15060364)
Supplement: Supplementary file 1 [file metabolites-15-00364-s001.zip › Figure S1_S2.pdf]

Plasma TMAO concentrations and gut microbiota composition in subjects with and without metabolic syndrome: results from pilot study

Mohammed E Hefni, Cornelia M Witthöft, Patrik Hellström, Ingegerd Johansson and Anders Esberg [3]

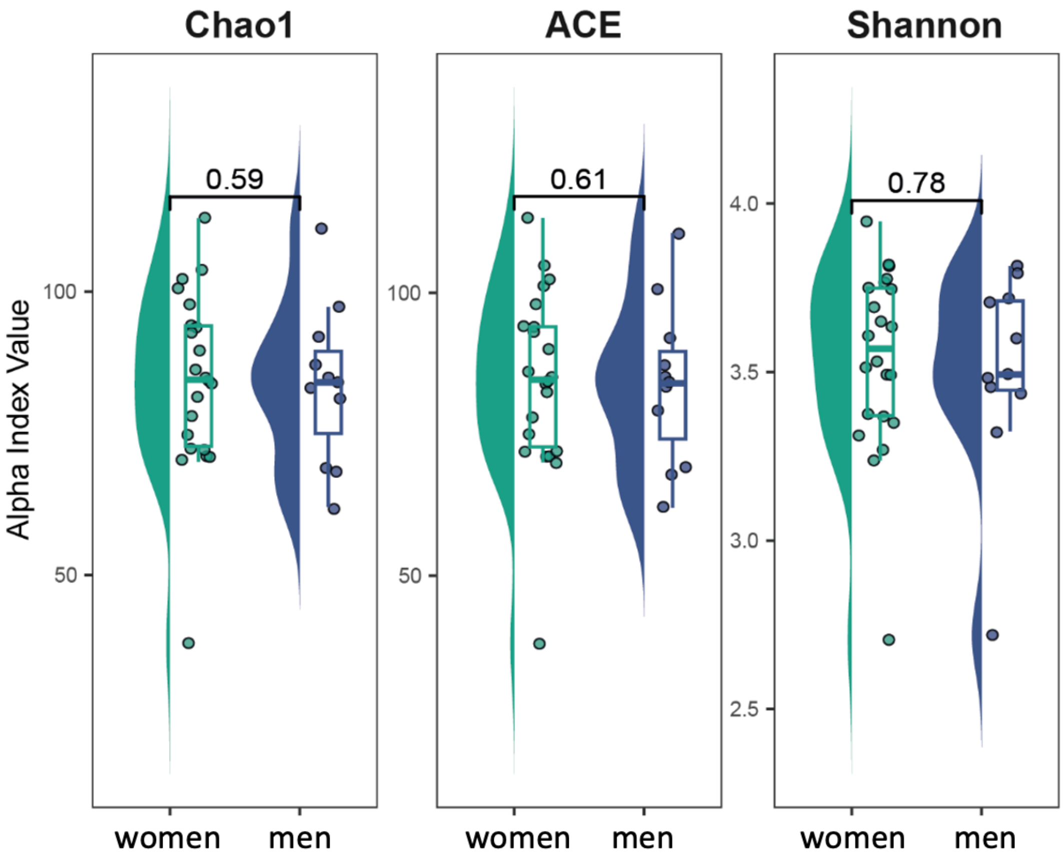

Figure S1. Raincloud plots showing alpha diversity (Chao1, ACE, Shannon indexes) by sex. Each plot combines a half-violin (distribution), a boxplot (median and interquartile range), and individual data points. The plots display microbial diversity in feces samples from women and men. P-values are places on top of the figure with statistical comparisons were performed using the Mann–Whitney U test.

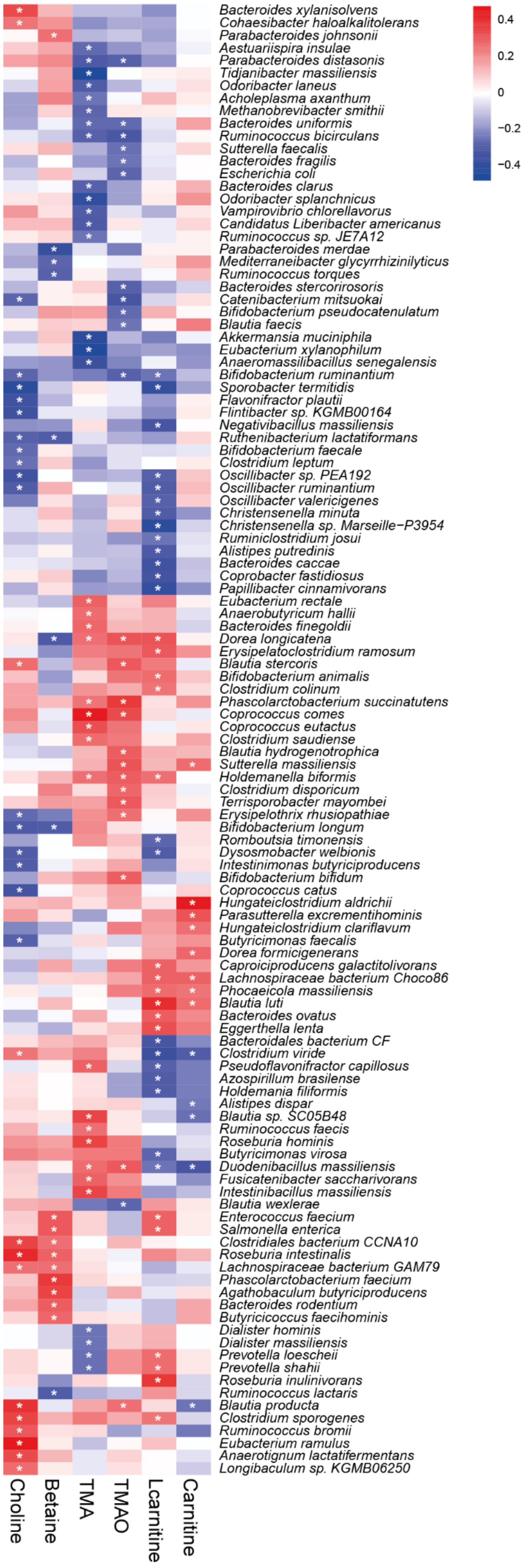

Figure S2. Heatmap illustrating the associations between microbial species and TMAO and related compounds. Each cell represents the strength and direction of the association between a given species (rows) and a clinical variable (columns), with color intensity indicating the effect size or correlation coefficient. Positive associations are shown in [red] and negative associations in [blue], allowing visual identification of patterns across multiple species and clinical traits. Hierarchical clustering was applied to both rows and columns to group species and clinical markers with similar association profiles.
