# Supplementary material for: Plasma TMAO Concentrations and Gut Microbiota Composition in Subjects with and Without Metabolic Syndrome: Results from Pilot Study
Source: Metabolites. 2025 May 30;15(6):364. doi: 10.3390/metabo15060364 (PMC12195445; doi:10.3390/metabo15060364)
Supplement: Supplementary file 1 [file metabolites-15-00364-s001.zip › Table S1 & S2.pdf]

Plasma TMAO concentrations and gut microbiota composition in subjects with and without metabolic syndrome.

Mohammed E Hefni, Cornelia M Witthöft, Patrik Hellström, Ingegerd Johansson and Anders Esberg

Table S1. Sequencing characteristics. Nanopore sequencing of 33 samples generate 3,493,134 quality-filtered EMU mapped reads. After rarefying the data to a consistent sampling depth of 28,900 reads per sample, the reads represented 9 phyla, 121 genera, and 232 species. Rarified number of sequences (n) and proportion (%) of the total number of reads, prevalence (n) and proportion (%) of participants are presented. Rarefying was done using the MicrobiotaProcess R package.

		Number fo reads		Prevalence	
**Class**	**Phylum**	n	%	n	%
Phyla	p_Firmicutes	559694	58.8%	33	100.0%
	p_Bacteroidetes	287986	30.3%	33	100.0%
	p_Actinobacteria	57292	6.0%	32	97.0%
	p_Verrucomicrobia	17673	1.9%	20	60.6%
	p_Proteobacteria	16389	1.7%	33	100.0%
	p_Tenericutes	7617	0.8%	15	45.5%
	p_Lentisphaerae	3502	0.4%	13	39.4%
	p_Melainabacteria	1484	0.2%	13	39.4%
	p_Euryarchaeota	347	0.0%	4	12.1%
Genera	g_Anaerobutyricum	15885	1.7%	33	100%
	g_Anaerostipes	13263	1.4%	33	100%
	g_Bacteroides	79013	8.3%	33	100%
	g_Blautia	92109	9.7%	33	100%
	g_Faecalibacterium	104172	10.9%	33	100%
	g_Fusicatenibacter	23652	2.5%	33	100%
	g_Phocaeicola	94281	9.9%	33	100%
	g_Ruminococcus	56731	6.0%	33	100%
	g_Alistipes	26164	2.7%	32	97%
	g_Coprococcus	18257	1.9%	32	97%
	g_Dorea	8684	0.9%	32	97%
	g_Eubacterium	23571	2.5%	32	97%
	g_Gemmiger	31646	3.3%	32	97%
	g_Roseburia	21999	2.3%	32	97%
	g_Agathobaculum	4512	0.5%	31	94%
	g_Dysosmobacter	12280	1.3%	31	94%
	g_Flintibacter	3641	0.4%	31	94%
	g_Bifidobacterium	41129	4.3%	30	91%
	g_Oscillibacter	14530	1.5%	30	91%
	g_Parabacteroides	14741	1.5%	30	91%
	g_Subdoligranulum	3179	0.3%	30	91%
	g_Collinsella	15314	1.6%	29	88%
	g_Cuneatibacter	4515	0.5%	29	88%
	g_Barnesiella	9432	1.0%	28	85%
	g_Lachnospiraceae	7532	0.8%	27	82%
	g_Odoribacter	3047	0.3%	27	82%
	g_Clostridium	6765	0.7%	26	79%

g_Hungatella	2403	0.3%	26	79%
g_Lachnospira	6191	0.7%	26	79%
g_Monoglobus	1842	0.2%	25	76%
g_Christensenella	11088	1.2%	24	73%
g_Dialister	10539	1.1%	23	70%
g_Enterocloster	2377	0.2%	22	67%
g_Lacrimispora	1339	0.1%	22	67%
g_Romboutsia	7725	0.8%	22	67%
g_Anaeromassilibacillus	3648	0.4%	21	64%
g_Lactobacillus	3182	0.3%	21	64%
g_Neglecta	2534	0.3%	21	64%
g_Akkermansia	17564	1.8%	20	61%
g_Faecalicatena	2328	0.2%	19	58%
g_Sutterella	6704	0.7%	19	58%
g_Erysipelotrichaceae	3248	0.3%	18	55%
g_Intestinimonas	2958	0.3%	18	55%
g_Phascolarctobacterium	3497	0.4%	15	45%
g_Sporobacter	1609	0.2%	15	45%
g_Eisenbergiella	1023	0.1%	14	42%
g_Flavonifractor	1564	0.2%	14	42%
g_Catabacter	1983	0.2%	13	39%
g_Colidextribacter	574	0.1%	13	39%
g_Vampirovibrio	1484	0.2%	13	39%
g_Victivallales	3216	0.3%	13	39%
g_Asteroleplasma	6109	0.6%	12	36%
g_Faecalimonas	1261	0.1%	12	36%
g_Intestinibacter	1960	0.2%	12	36%
g_Papillibacter	1422	0.1%	12	36%
g_Coprobacter	768	0.1%	11	33%
g_Desulfovibrio	1835	0.2%	11	33%
g_Butyricicoccus	545	0.1%	10	30%
g_Clostridiales	225	0.0%	10	30%
g_Prevotella	40664	4.3%	10	30%
g_Duodenibacillus	1895	0.2%	9	27%
g_Streptococcus	1265	0.1%	9	27%
g_Turicibacter	832	0.1%	9	27%
g_Butyricimonas	1323	0.1%	8	24%
g_Holdemanella	2581	0.3%	8	24%
g_Paraprevotella	1700	0.2%	8	24%
g_Parasutterella	1528	0.2%	8	24%
g_Pseudoflavonifractor	143	0.0%	8	24%
g_Anaerotignum	46	0.0%	7	21%
g_Erysipelothrix	956	0.1%	7	21%
g_Hungateiclostridium	513	0.1%	7	21%
g_Muribaculum	2188	0.2%	7	21%
g_Senegalimassilia	307	0.0%	7	21%
g_Terrisporobacter	1002	0.1%	7	21%
g_Cohaesibacter	116	0.0%	6	18%

	g_Escherichia	3183	0.3%	6	18%
	g_Kiloniella	432	0.0%	6	18%
	g_Longicatena	585	0.1%	6	18%
	g_Anaeroplasma	1240	0.1%	5	15%
	g_Anaerotruncus	425	0.0%	5	15%
	g_Butyrivibrio	1864	0.2%	5	15%
	g_Duncaniella	1788	0.2%	5	15%
	g_Pseudoclostridium	486	0.1%	5	15%
	g_Raoultibacter	343	0.0%	5	15%
	g_Tidjanibacter	260	0.0%	5	15%
	g_Aestuariispira	166	0.0%	4	12%
	g_Bacteroidales	7778	0.8%	4	12%
	g_Hespellia	28	0.0%	4	12%
	g_Massiliprevotella	4606	0.5%	4	12%
	g_Methanobrevibacter	347	0.0%	4	12%
	g_Negativibacillus	372	0.0%	4	12%
	g_Ruminiclostridium	63	0.0%	4	12%
	g_Ruthenibacterium	480	0.1%	4	12%
	g_Victivallis	286	0.0%	4	12%
	g_Adlercreutzia	132	0.0%	3	9%
	g_Amedibacillus	60	0.0%	3	9%
	g_Candidatus	59	0.0%	3	9%
	g_Caproiciproducens	99	0.0%	3	9%
	g_Eggerthella	67	0.0%	3	9%
	g_Falcatimonas	22	0.0%	3	9%
	g_Intestinibacillus	55	0.0%	3	9%
	g_Longibaculum	11	0.0%	3	9%
	g_Tepidibaculum	150	0.0%	3	9%
	g_Acholeplasma	268	0.0%	2	6%
	g_Azospirillum	458	0.0%	2	6%
	g_Catenibacterium	464	0.0%	2	6%
	g_Coraliomargarita	109	0.0%	2	6%
	g_Culturomica	284	0.0%	2	6%
	g_Enterococcus	2137	0.2%	2	6%
	g_Erysipelatoclostridium	34	0.0%	2	6%
	g_Fournierella	40	0.0%	2	6%
	g_Holdemania	7	0.0%	2	6%
	g_Marseilla	106	0.0%	2	6%
	g_Mediterraneibacter	124	0.0%	2	6%
	g_Mitsuokella	412	0.0%	2	6%
	g_Oxalobacter	3	0.0%	2	6%
	g_Phocea	103	0.0%	2	6%
	g_Salmonella	10	0.0%	2	6%
	g_Faecalitalea	64	0.0%	1	3%
	g_Frisingicoccus	84	0.0%	1	3%
	g_Lachnoclostridium	42	0.0%	1	3%
Species	s_Anaerobutyricum_hallii	15885	1.7%	33	100.0%

s_Anaerostipes_hadrus	13263	1.4%	33	100.0%
s_Blautia_luti	14369	1.5%	33	100.0%
s_Blautia_sp._SC05B48	29240	3.1%	33	100.0%
s_Blautia_wexlerae	27556	2.9%	33	100.0%
s_Faecalibacterium_prausnitzii	104172	10.9%	33	100.0%
s_Fusicatenibacter_saccharivorans	23652	2.5%	33	100.0%
s_Phocaeicola_dorei	29954	3.1%	33	100.0%
s_Dorea_longicatena	8240	0.9%	32	97.0%
s_Gemmiger_formicilis	31646	3.3%	32	97.0%
s_Agathobaculum_butyriciproducens	4512	0.5%	31	93.9%
s_Bacteroides_uniformis	25180	2.6%	31	93.9%
s_Blautia_faecis	9157	1.0%	31	93.9%
s_Coprococcus_comes	7997	0.8%	31	93.9%
s_Dysosmobacter_welbionis	12280	1.3%	31	93.9%
s_Flintibacter_sp._KGMB00164	3641	0.4%	31	93.9%
s_Ruminococcus_faecis	10474	1.1%	31	93.9%
s_Alistipes_putredinis	10967	1.2%	30	90.9%
s_Oscillibacter_valericigenes	9044	1.0%	30	90.9%
s_Roseburia_hominis	5744	0.6%	30	90.9%
s_Subdoligranulum_variabile	3179	0.3%	30	90.9%
s_Blautia_obeum	6110	0.6%	29	87.9%
s_Collinsella_aerofaciens	15314	1.6%	29	87.9%
s_Cuneatibacter_caecimuris	4515	0.5%	29	87.9%
s_Phocaeicola_vulgatus	48927	5.1%	29	87.9%
s_Bacteroides_ovatus	9918	1.0%	28	84.8%
s_Barnesiella_intestinihominis	9432	1.0%	28	84.8%
s_Eubacterium_rectale	19531	2.1%	28	84.8%
s_Oscillibacter_ruminantium	4647	0.5%	28	84.8%
s_Lachnospiraceae_bacterium_GAM79	7527	0.8%	27	81.8%
s_Parabacteroides_distasonis	5991	0.6%	27	81.8%
s_Roseburia_faecis	7558	0.8%	27	81.8%
s_Ruminococcus_gnavus	5687	0.6%	27	81.8%
s_Bifidobacterium_adolescentis	24720	2.6%	26	78.8%
s_Coprococcus_catus	2424	0.3%	26	78.8%
s_Hungatella_hathewayi	2382	0.3%	26	78.8%
s_Lachnospira_eligens	6137	0.6%	26	78.8%
s_Odoribacter_splanchnicus	2670	0.3%	26	78.8%
s_Ruminococcus_bromii	20064	2.1%	26	78.8%
s_Bifidobacterium_longum	11702	1.2%	25	75.8%
s_Monoglobus_pectinilyticus	1842	0.2%	25	75.8%
s_Blautia_producta	1926	0.2%	24	72.7%
s_Roseburia_intestinalis	5452	0.6%	24	72.7%
s_Blautia_stercoris	2519	0.3%	23	69.7%
s_Christensenella_sp._Marseille-P3954	5946	0.6%	23	69.7%
s_Eubacterium_coprostanoligenes	1708	0.2%	23	69.7%
s_Ruminococcus_bicirculans	8996	0.9%	23	69.7%
s_Bacteroides_thetaiotaomicron	2790	0.3%	22	66.7%
s_Coprococcus_eutactus	7836	0.8%	22	66.7%

s_Enterocloster_bolteae	2375	0.2%	22	66.7%
s_Romboutsia_timonensis	7725	0.8%	22	66.7%
s_Roseburia_inulinivorans	3245	0.3%	22	66.7%
s_Ruminococcus_torques	3822	0.4%	22	66.7%
s_Alistipes_shahii	2791	0.3%	21	63.6%
s_Anaeromassilibacillus_senegalensis	3648	0.4%	21	63.6%
s_Lactobacillus_rogosae	3182	0.3%	21	63.6%
s_Neglecta_timonensis	2534	0.3%	21	63.6%
s_Parabacteroides_merdae	6622	0.7%	21	63.6%
s_Akkermansia_muciniphila	17564	1.8%	20	60.6%
s_Bacteroides_caccae	9230	1.0%	20	60.6%
s_Bacteroides_cellulosilyticus	7230	0.8%	19	57.6%
s_Oscillibacter_sp._PEA192	839	0.1%	19	57.6%
s_Alistipes_onderdonkii	6665	0.7%	18	54.5%
s_Clostridium_saudiense	5465	0.6%	18	54.5%
s_Erysipelotrichaceae_bacterium_GAM147	2654	0.3%	18	54.5%
s_Intestinimonas_butyriciproducens	2958	0.3%	18	54.5%
s_Lacrimispora_saccharolytica	978	0.1%	18	54.5%
s_Ruminococcus_champanellensis	2412	0.3%	18	54.5%
s_Alistipes_finegoldii	1896	0.2%	17	51.5%
s_Bacteroides_xylanisolvens	1294	0.1%	17	51.5%
s_Blautia_glucerasea	1182	0.1%	17	51.5%
s_Dialister_invisus	7752	0.8%	17	51.5%
s_Faecalicatena_fissicatena	2254	0.2%	17	51.5%
s_Ruminococcus_callidus	2723	0.3%	17	51.5%
s_Alistipes_communis	1167	0.1%	16	48.5%
s_Bifidobacterium_faecale	1403	0.1%	16	48.5%
s_Eubacterium_ramulus	992	0.1%	16	48.5%
s_Ruminococcus_lactaris	1536	0.2%	16	48.5%
s_Sutterella_wadsworthensis	5494	0.6%	16	48.5%
s_Sporobacter_termitidis	1609	0.2%	15	45.5%
s_Eisenbergiella_tayi	1023	0.1%	14	42.4%
s_Flavonifractor_plautii	1564	0.2%	14	42.4%
s_Phocaeicola_massiliensis	12077	1.3%	14	42.4%
s_Catabacter_hongkongensis	1983	0.2%	13	39.4%
s_Colidextribacter_massiliensis	574	0.1%	13	39.4%
s_Parabacteroides_sp._CT06	1088	0.1%	13	39.4%
s_Vampirovibrio_chlorellavorus	1484	0.2%	13	39.4%
s_Victivallales_bacterium_CCUG_44730	3216	0.3%	13	39.4%
s_Asteroleplasma_anaerobium	6109	0.6%	12	36.4%
s_Faecalimonas_umbilicata	1261	0.1%	12	36.4%
s_Intestinibacter_bartlettii	1960	0.2%	12	36.4%
s_Papillibacter_cinnamivorans	1422	0.1%	12	36.4%
s_Phascolarctobacterium_faecium	2266	0.2%	12	36.4%
s_Bacteroides_stercoris	4956	0.5%	11	33.3%
s_Christensenella_massiliensis	2947	0.3%	11	33.3%
s_Coprobacter_fastidiosus	768	0.1%	11	33.3%
s_Bacteroides_eggerthii	3821	0.4%	10	30.3%

s_Bacteroides_fragilis	5602	0.6%	10	30.3%
s_Butyricicoccus_faecihominis	545	0.1%	10	30.3%
s_Lacrimispora_amygdalina	361	0.0%	10	30.3%
s_Prevotella_copri	38454	4.0%	10	30.3%
s_Bacteroides_intestinalis	1870	0.2%	9	27.3%
s_Desulfovibrio_piger	1729	0.2%	9	27.3%
s_Dorea_formicigenerans	444	0.0%	9	27.3%
s_Duodenibacillus_massiliensis	1895	0.2%	9	27.3%
s_Eubacterium_siraeum	936	0.1%	9	27.3%
s_Eubacterium_ventriosum	145	0.0%	9	27.3%
s_Holdemanella_biformis	2581	0.3%	8	24.2%
s_Paraprevotella_clara	1700	0.2%	8	24.2%
s_Parasutterella_excrementihominis	1528	0.2%	8	24.2%
s_Bacteroides_faecis	2577	0.3%	7	21.2%
s_Christensenella_minuta	1432	0.2%	7	21.2%
s_Clostridiales_bacterium	78	0.0%	7	21.2%
s_Clostridium_leptum	537	0.1%	7	21.2%
s_Erysipelothrix_rhusiopathiae	956	0.1%	7	21.2%
s_Muribaculum_intestinale	2188	0.2%	7	21.2%
s_Phascolarctobacterium_succinatutens	1231	0.1%	7	21.2%
s_Pseudoflavonifractor_phocaeensis	94	0.0%	7	21.2%
s_Senegalimassilia_anaerobia	307	0.0%	7	21.2%
s_Streptococcus_salivarius	846	0.1%	7	21.2%
s_Alistipes_dispar	2161	0.2%	6	18.2%
s_Clostridium_spiroforme	287	0.0%	6	18.2%
s_Cohaesibacter_haloalkalitolerans	116	0.0%	6	18.2%
s_Escherichia_coli	3183	0.3%	6	18.2%
s_Eubacterium_xylanophilum	194	0.0%	6	18.2%
s_Faecalicatena_contorta	68	0.0%	6	18.2%
s_Kiloniella_majae	432	0.0%	6	18.2%
s_Longicatena_caecimuris	585	0.1%	6	18.2%
s_Ruminococcus_sp._JE7A12	1017	0.1%	6	18.2%
s_Turicibacter_sp._H121	649	0.1%	6	18.2%
s_Alistipes_indistinctus	367	0.0%	5	15.2%
s_Alistipes_senegalensis	150	0.0%	5	15.2%
s_Anaeroplasma_varium	744	0.1%	5	15.2%
s_Anaerotignum_faecicola	41	0.0%	5	15.2%
s_Anaerotruncus_rubiinfantis	425	0.0%	5	15.2%
s_Bacteroides_clarus	1614	0.2%	5	15.2%
s_Bifidobacterium_animalis	830	0.1%	5	15.2%
s_Bifidobacterium_ruminantium	234	0.0%	5	15.2%
s_Butyrivibrio_crossotus	1864	0.2%	5	15.2%
s_Christensenella_timonensis	763	0.1%	5	15.2%
s_Clostridium_colinum	202	0.0%	5	15.2%
s_Duncaniella_sp._B8	1788	0.2%	5	15.2%
s_Erysipelotrichaceae_bacterium_I46	594	0.1%	5	15.2%
s_Hungateiclostridium_clariflavum	286	0.0%	5	15.2%
s_Parabacteroides_goldsteinii	327	0.0%	5	15.2%

s_Parabacteroides_johnsonii	713	0.1%	5	15.2%
s_Pseudoclostridium_thermosuccinogenes	486	0.1%	5	15.2%
s_Raoultibacter_timonensis	343	0.0%	5	15.2%
s_Terrisporobacter_mayombei	710	0.1%	5	15.2%
s_Terrisporobacter_petrolearius	292	0.0%	5	15.2%
s_Tidjanibacter_massiliensis	260	0.0%	5	15.2%
s_Aestuariispira_insulae	166	0.0%	4	12.1%
s_Anaeroplasma_abactoclasticum	496	0.1%	4	12.1%
s_Bacteroidales_bacterium_CF	7778	0.8%	4	12.1%
s_Bacteroides_finegoldii	867	0.1%	4	12.1%
s_Bacteroides_salyersiae	557	0.1%	4	12.1%
s_Clostridiales_bacterium_CCNA10	147	0.0%	4	12.1%
s_Eubacterium_oxidoreducens	65	0.0%	4	12.1%
s_Hespellia_stercorisuis	28	0.0%	4	12.1%
s_Hungateiclostridium_aldrichii	227	0.0%	4	12.1%
s_Hungatella_xylanolytica	21	0.0%	4	12.1%
s_Massiliprevotella_massiliensis	4606	0.5%	4	12.1%
s_Methanobrevibacter_smithii	347	0.0%	4	12.1%
s_Negativibacillus_massiliensis	372	0.0%	4	12.1%
s_Ruminiclostridium_josui	63	0.0%	4	12.1%
s_Ruthenibacterium_lactatiformans	480	0.1%	4	12.1%
s_Streptococcus_thermophilus	419	0.0%	4	12.1%
s_Sutterella_massiliensis	969	0.1%	4	12.1%
s_Turicibacter_sanguinis	183	0.0%	4	12.1%
s_Victivallis_vadensis	286	0.0%	4	12.1%
s_Adlercreutzia_equolifaciens	132	0.0%	3	9.1%
s_Amedibacillus_dolichus	60	0.0%	3	9.1%
s_Bacteroides_koreensis	126	0.0%	3	9.1%
s_Bacteroides_rodentium	94	0.0%	3	9.1%
s_Bacteroides_stercorirosoris	1086	0.1%	3	9.1%
s_Bifidobacterium_bifidum	571	0.1%	3	9.1%
s_Bifidobacterium_pseudocatenulatum	1450	0.2%	3	9.1%
s_Butyricimonas_paravirosa	11	0.0%	3	9.1%
s_Butyricimonas_virosa	704	0.1%	3	9.1%
s_Candidatus_Liberibacter_americanus	59	0.0%	3	9.1%
s_Clostridium_disporicum	118	0.0%	3	9.1%
s_Desulfovibrio_desulfuricans	106	0.0%	3	9.1%
s_Dialister_hominis	552	0.1%	3	9.1%
s_Dialister_massiliensis	324	0.0%	3	9.1%
s_Dialister_succinatiphilus	1911	0.2%	3	9.1%
s_Eggerthella_lenta	67	0.0%	3	9.1%
s_Falcatimonas_natans	22	0.0%	3	9.1%
s_Intestinibacillus_massiliensis	55	0.0%	3	9.1%
s_Longibaculum_sp._KGMB06250	11	0.0%	3	9.1%
s_Phocaeicola_coprocola	1996	0.2%	3	9.1%
s_Tepidibaculum_saccharolyticum	150	0.0%	3	9.1%
s_Acholeplasma_axanthum	268	0.0%	2	6.1%
s_Anaerotignum_lactatifermentans	5	0.0%	2	6.1%

s_Azospirillum_brasilense	458	0.0%	2	6.1%
s_Bacteroides_nordii	44	0.0%	2	6.1%
s_Bacteroides_pectinophilus	157	0.0%	2	6.1%
s_Bifidobacterium_catenulatum	219	0.0%	2	6.1%
s_Blautia_hydrogenotrophica	39	0.0%	2	6.1%
s_Butyricimonas_faecalis	320	0.0%	2	6.1%
s_Butyricimonas_faecihominis	288	0.0%	2	6.1%
s_Caproiciproducens_galactitolivorans	98	0.0%	2	6.1%
s_Catenibacterium_mitsuokai	464	0.0%	2	6.1%
s_Clostridium_sp._BNL1100	38	0.0%	2	6.1%
s_Clostridium_sporogenes	69	0.0%	2	6.1%
s_Clostridium_viride	49	0.0%	2	6.1%
s_Coraliomargarita_akajimensis	109	0.0%	2	6.1%
s_Culturomica_massiliensis	284	0.0%	2	6.1%
s_Enterocloster_clostridioformis	2	0.0%	2	6.1%
s_Enterococcus_faecium	2137	0.2%	2	6.1%
s_Erysipelatoclostridium_ramosum	34	0.0%	2	6.1%
s_Fournierella_massiliensis	40	0.0%	2	6.1%
s_Holdemania_filiformis	7	0.0%	2	6.1%
s_Lachnospira_pectinoschiza	54	0.0%	2	6.1%
s_Marseilla_massiliensis	106	0.0%	2	6.1%
s_Mediterraneibacter_glycyrrhizinilyticus	124	0.0%	2	6.1%
s_Mitsuokella_jalaludinii	412	0.0%	2	6.1%
s_Odoribacter_laneus	377	0.0%	2	6.1%
s_Oxalobacter_formigenes	3	0.0%	2	6.1%
s_Phocaeicola_plebeius	1327	0.1%	2	6.1%
s_Phocea_massiliensis	103	0.0%	2	6.1%
s_Prevotella_loescheii	1234	0.1%	2	6.1%
s_Prevotella_shahii	976	0.1%	2	6.1%
s_Pseudoflavonifractor_capillosus	49	0.0%	2	6.1%
s_Salmonella_enterica	10	0.0%	2	6.1%
s_Sutterella_faecalis	241	0.0%	2	6.1%
s_Blautia_hominis	11	0.0%	1	3.0%
s_Caproiciproducens_sp._NJN-50	1	0.0%	1	3.0%
s_Faecalicatena_orotica	6	0.0%	1	3.0%
s_Faecalitalea_cylindroides	64	0.0%	1	3.0%
s_Frisingicoccus_caecimuris	84	0.0%	1	3.0%
s_Lachnoclostridium_sp._YL32	42	0.0%	1	3.0%
s_Lachnospiraceae_bacterium_Choco86	5	0.0%	1	3.0%

Plasma TMAO concentrations and gut microbiota composition in subjects with and without metabolic syndrome.

Mohammed E Hefni, Cornelia M Witthöft, Patrik Hellström, Ingegerd Johansson and Anders Esberg

Table S2.Maaslin3 (Microbiome Multivariable Associations with Linear Models, R package) results determine multivariable associations between metabolic and non-metabolic syndromes and the abundance and prevalence of species while accounting for covariates such as the number of reads, age, and sex.

Species	Meta	coef	stderr	pval_indiv	error	model	N	N_not_zero
s_Ruminococcus_torques	Met	2.56	0.78	**0.0041**	NA	abundance	33	22
s_Blautia_glucerasea	Met	(2.58)	0.95	**0.0067**	NA	prevalence	33	17
s_Ruminococcus_champanellensis	Met	(2.18)	0.94	**0.0203**	NA	prevalence	33	19
s_Kiloniella_majae	Met	(18.53)	0.53	**0.0217**	NA	abundance	33	6
s_Ruminococcus_bicirculans	Met	(2.40)	1.07	**0.0247**	NA	prevalence	33	23
s_Monoglobus_pectinilyticus	Met	(2.50)	1.14	**0.0278**	NA	prevalence	33	25
s_Lachnospiraceae_bacterium_GAM79	Met	(2.37)	0.86	**0.0307**	NA	abundance	33	27
s_Blautia_stercoris	Met	(2.57)	1.22	**0.0350**	NA	prevalence	33	24
s_Bifidobacterium_ruminantium	Met	5.93	0.17	**0.0381**	NA	abundance	33	6
s_Roseburia_intestinalis	Met	(2.16)	1.07	**0.0433**	NA	prevalence	33	25
s_Christensenella_sp._Marseille.P3954	Met	(1.92)	0.95	**0.0442**	NA	prevalence	33	24
s_Desulfovibrio_piger	Met	(2.72)	1.35	**0.0442**	NA	prevalence	33	9
s_Coprococcus_eutactus	Met	(1.90)	0.96	**0.0473**	NA	prevalence	33	22
s_Bacteroides_fragilis	Met	3.23	1.30	**0.0488**	NA	abundance	33	10
s_Blautia_producta	Met	(1.96)	1.00	**0.0496**	NA	prevalence	33	25
s_Faecalicatena_fissicatena	Met	(1.77)	0.93	0.0579	NA	prevalence	33	17
s_Ruminococcus_lactaris	Met	(3.32)	1.43	0.0603	NA	abundance	33	16
s_Bifidobacterium_adolescentis	Met	(1.83)	0.98	0.0613	NA	prevalence	33	26
s_Butyricicoccus_faecihominis	Met	(1.85)	1.01	0.0682	NA	prevalence	33	10
s_Ruminococcus_gnavus	Met	1.46	0.85	0.0716	NA	abundance	33	28
s_Cohaesibacter_haloalkalitolerans	Met	(2.45)	1.37	0.0737	NA	prevalence	33	6
s_Eubacterium_rectale	Met	1.54	0.90	0.0759	NA	abundance	33	28

s_Terrisporobacter_mayombei	Met	(7.14)	0.78	0.0775	NA	abundance	33	6
s_Ruminococcus_callidus	Met	(1.44)	0.82	0.0784	NA	prevalence	33	17
s_Alistipes_communis	Met	(1.53)	0.87	0.0794	NA	prevalence	33	17
s_Coprococcus_catus	Met	1.41	0.85	0.0817	NA	abundance	33	27
s_Eubacterium_ventriosum	Met	2.28	1.07	0.0872	NA	abundance	33	9
s_Duncaniella_sp._B8	Met	(2.58)	1.53	0.0919	NA	prevalence	33	5
s_Dialister_invisus	Met	(2.18)	1.01	0.0931	NA	abundance	33	17
s_Hungatella_hathewayi	Met	(1.52)	0.66	0.1013	NA	abundance	33	26
s_Ruminococcus_champanellensis	Met	(2.93)	1.51	0.1042	NA	abundance	33	19
s_Romboutsia_timonensis	Met	(1.32)	0.82	0.1056	NA	prevalence	33	22
s_Eubacterium_oxidoreducens	Met	(2.28)	1.42	0.1076	NA	prevalence	33	4
s_Alistipes_shahii	Met	(1.35)	0.84	0.1093	NA	prevalence	33	22
s_Blautia_obeum	Met	(1.33)	0.57	0.1110	NA	abundance	33	29
s_Monoglobus_pectinilyticus	Met	(1.88)	0.92	0.1116	NA	abundance	33	25
s_Lachnospira_eligens	Met	(1.65)	1.05	0.1159	NA	prevalence	33	26
s_Phascolarctobacterium_faecium	Met	1.34	0.86	0.1186	NA	prevalence	33	12
s_Streptococcus_salivarius	Met	(3.38)	1.16	0.1213	NA	abundance	33	7
s_Dorea_formicigenerans	Met	2.18	1.20	0.1222	NA	abundance	33	9
s_Alistipes_senegalensis	Met	(1.62)	1.06	0.1240	NA	prevalence	33	7
s_Lacrimispora_saccharolytica	Met	(1.92)	0.97	0.1246	NA	abundance	33	18
s_Vampirovibrio_chlorellavorus	Met	(1.33)	0.87	0.1248	NA	prevalence	33	13
s_Agathobaculum_butyriciproducens	Met	(1.47)	0.69	0.1248	NA	abundance	33	31
s_Bacteroides_ovatus	Met	2.12	1.39	0.1263	NA	prevalence	33	28
s_Alistipes_onderdonkii	Met	2.07	1.39	0.1268	NA	abundance	33	18
s_Roseburia_hominis	Met	(1.36)	0.63	0.1307	NA	abundance	33	30
s_Muribaculum_intestinale	Met	(1.59)	1.05	0.1322	NA	prevalence	33	7
s_Clostridium_saudiense	Met	(1.38)	0.93	0.1361	NA	prevalence	33	18
s_Anaerostipes_hadrus	Met	0.53	0.39	0.1371	NA	abundance	33	33
s_Massiliprevotella_massiliensis	Met	(2.07)	1.40	0.1381	NA	prevalence	33	4
s_Coprobacter_fastidiosus	Met	1.26	0.85	0.1382	NA	prevalence	33	11

s_Eubacterium_ramulus	Met	(1.28)	0.87	0.1425	NA	prevalence	33	17
s_Clostridiales_bacterium	Met	(2.02)	1.38	0.1441	NA	prevalence	33	7
s_Butyricimonas_paravirosa	Met	1.68	1.15	0.1441	NA	prevalence	33	4
s_Lachnospiraceae_bacterium_GAM79	Met	(1.49)	1.02	0.1455	NA	prevalence	33	27
s_Hungateiclostridium_aldrichii	Met	1.43	0.98	0.1459	NA	prevalence	33	5
s_Escherichia_coli	Met	1.39	0.96	0.1459	NA	prevalence	33	7
s_Roseburia_hominis	Met	(2.18)	1.50	0.1478	NA	prevalence	33	30
s_Parabacteroides_distasonis	Met	(1.45)	1.00	0.1478	NA	prevalence	33	27
s_Eubacterium_ramulus	Met	1.72	1.24	0.1494	NA	abundance	33	17
s_Bacteroides_thetaiotaomicron	Met	1.65	1.23	0.1506	NA	abundance	33	23
s_Enterocloster_clostridioformis	Met	2.01	1.40	0.1513	NA	prevalence	33	3
s_Phocaeicola_vulgatus	Met	(1.62)	1.13	0.1532	NA	prevalence	33	29
s_Victivallis_vadensis	Met	(1.97)	1.38	0.1546	NA	prevalence	33	4
s_Tidjanibacter_massiliensis	Met	(7.40)	1.75	0.1555	NA	abundance	33	6
s_Butyricimonas_faecihominis	Met	2.05	1.45	0.1571	NA	prevalence	33	2
s_Alistipes_indistinctus	Met	(1.91)	1.37	0.1639	NA	prevalence	33	7
s_Streptococcus_thermophilus	Met	(1.86)	1.35	0.1659	NA	prevalence	33	4
s_Oscillibacter_valericigenes	Met	(1.22)	0.60	0.1678	NA	abundance	33	30
s_Parasutterella_excrementihominis	Met	1.17	0.85	0.1689	NA	prevalence	33	10
s_Bacteroides_fragilis	Met	1.23	0.90	0.1714	NA	prevalence	33	10
s_Ruminiclostridium_josui	Met	1.46	1.08	0.1750	NA	prevalence	33	4
s_Clostridium_sporogenes	Met	1.31	0.96	0.1751	NA	prevalence	33	5
s_Ruminococcus_gnavus	Met	(1.42)	1.06	0.1790	NA	prevalence	33	28
s_Anaerotruncus_rubiinfantis	Met	(1.79)	1.35	0.1865	NA	prevalence	33	5
s_Sporobacter_termitidis	Met	(1.14)	0.86	0.1873	NA	prevalence	33	17
s_Butyrivibrio_crossotus	Met	(1.82)	1.39	0.1889	NA	prevalence	33	5
s_Butyricicoccus_faecihominis	Met	(3.29)	1.98	0.1896	NA	abundance	33	10
s_Blautia_faecis	Met	0.70	0.63	0.1922	NA	abundance	33	31
s_Tepidibaculum_saccharolyticum	Met	(1.83)	1.40	0.1931	NA	prevalence	33	3
s_Alistipes_senegalensis	Met	(6.45)	3.22	0.1956	NA	abundance	33	7

s_Prevotella_loescheii	Met	1.80	1.41	0.1999	NA	prevalence	33	2
s_Bacteroides_cellulosilyticus	Met	1.81	1.51	0.2043	NA	abundance	33	19
s_Bacteroides_koreensis	Met	1.58	1.25	0.2069	NA	prevalence	33	3
s_Holdemanella_biformis	Met	4.29	3.01	0.2074	NA	abundance	33	9
s_Victivallales_bacterium_CCUG_44730	Met	(2.88)	1.94	0.2160	NA	abundance	33	13
s_Alistipes_shahii	Met	1.22	1.11	0.2176	NA	abundance	33	22
s_Bifidobacterium_animalis	Met	(1.40)	1.14	0.2179	NA	prevalence	33	5
s_Blautia_hominis	Met	1.65	1.34	0.2185	NA	prevalence	33	2
s_Oscillibacter_ruminantium	Met	(1.31)	1.07	0.2213	NA	prevalence	33	28
s_Lacrimispora_amygdalina	Met	1.86	1.51	0.2292	NA	abundance	33	10
s_Eisenbergiella_tayi	Met	0.98	0.82	0.2370	NA	prevalence	33	15
s_Bacteroides_salyersiae	Met	(1.30)	1.10	0.2374	NA	prevalence	33	5
s_Bifidobacterium_pseudocatenulatum	Met	1.53	1.30	0.2376	NA	prevalence	33	3
s_Mediterraneibacter_glycyrrhizinilyticus	Met	1.57	1.33	0.2380	NA	prevalence	33	2
s_Victivallales_bacterium_CCUG_44730	Met	(0.98)	0.83	0.2386	NA	prevalence	33	13
s_Romboutsia_timonensis	Met	(1.68)	1.15	0.2468	NA	abundance	33	22
s_Intestinimonas_butyriciproducens	Met	(2.17)	1.56	0.2475	NA	abundance	33	19
s_Clostridium_leptum	Met	1.19	1.03	0.2483	NA	prevalence	33	7
s_Prevotella_shahii	Met	1.47	1.28	0.2496	NA	prevalence	33	3
s_Streptococcus_salivarius	Met	(1.07)	0.94	0.2548	NA	prevalence	33	7
s_Blautia_obeum	Met	1.56	1.37	0.2557	NA	prevalence	33	29
s_Sutterella_faecalis	Met	(1.60)	1.42	0.2578	NA	prevalence	33	2
s_Bacteroides_ovatus	Met	0.54	0.62	0.2724	NA	abundance	33	28
s_Parabacteroides_sp._CT06	Met	1.65	1.59	0.2745	NA	abundance	33	13
s_Enterococcus_faecium	Met	1.38	1.28	0.2803	NA	prevalence	33	2
s_Salmonella_enterica	Met	1.38	1.28	0.2803	NA	prevalence	33	2
s_Lacrimispora_saccharolytica	Met	(0.88)	0.83	0.2849	NA	prevalence	33	18
s_Intestinimonas_butyriciproducens	Met	(0.91)	0.86	0.2857	NA	prevalence	33	19
s_Haemophilus_parainfluenzae	Met	(1.56)	1.46	0.2862	NA	prevalence	33	2
s_Bacteroides_clarus	Met	(1.27)	1.19	0.2878	NA	prevalence	33	5

s_Parabacteroides_distasonis	Met	0.48	0.61	0.2999	NA	abundance	33	27
s_Asteroleplasma_anaerobium	Met	3.07	2.96	0.3005	NA	abundance	33	12
s_Bifidobacterium_adolescentis	Met	0.68	0.83	0.3020	NA	abundance	33	26
s_Bacteroidales_bacterium_CF	Met	(1.40)	1.38	0.3109	NA	prevalence	33	4
s_Lactobacillus_rogosae	Met	1.12	1.28	0.3137	NA	abundance	33	21
s_Parabacteroides_merdae	Met	(0.80)	0.80	0.3147	NA	prevalence	33	21
s_Muribaculum_intestinale	Met	(7.81)	5.78	0.3215	NA	abundance	33	7
s_Eubacterium_coprostanoligenes	Met	(0.89)	0.90	0.3244	NA	prevalence	33	24
s_Eubacterium_xylanophilum	Met	(0.90)	0.92	0.3244	NA	prevalence	33	9
s_Phocea_massiliensis	Met	(1.38)	1.40	0.3248	NA	prevalence	33	2
s_Lachnospira_pectinoschiza	Met	(1.39)	1.42	0.3276	NA	prevalence	33	2
s_Blautia_stercoris	Met	0.93	1.14	0.3280	NA	abundance	33	24
s_Clostridium_sp._BNL1100	Met	1.13	1.16	0.3302	NA	prevalence	33	3
s_Parabacteroides_sp._CT06	Met	0.75	0.78	0.3333	NA	prevalence	33	13
s_Erysipelatoclostridium_ramosum	Met	1.21	1.27	0.3386	NA	prevalence	33	2
s_Asteroleplasma_anaerobium	Met	(0.80)	0.86	0.3525	NA	prevalence	33	12
s_Barnesiella_intestinihominis	Met	0.50	0.73	0.3554	NA	abundance	33	28
s_Lachnospiraceae_bacterium_Choco86	Met	(1.45)	1.57	0.3561	NA	prevalence	33	2
s_Hungateiclostridium_clariflavum	Met	0.81	0.89	0.3596	NA	prevalence	33	7
s_Dialister_invisus	Met	(0.70)	0.77	0.3670	NA	prevalence	33	17
s_Intestinibacter_bartlettii	Met	1.32	1.60	0.3677	NA	abundance	33	12
s_Blautia_wexlerae	Met	0.29	0.50	0.3689	NA	abundance	33	33
s_Bacteroides_nordii	Met	1.34	1.50	0.3697	NA	prevalence	33	2
s_Erysipelotrichaceae_bacterium_I46	Met	0.92	1.03	0.3703	NA	prevalence	33	5
s_Lacrimispora_amygdalina	Met	(0.79)	0.88	0.3704	NA	prevalence	33	10
s_Odoribacter_laneus	Met	(1.28)	1.44	0.3743	NA	prevalence	33	2
s_Oscillibacter_ruminantium	Met	0.49	0.75	0.3770	NA	abundance	33	28
s_Fournierella_massiliensis	Met	(1.31)	1.49	0.3802	NA	prevalence	33	2
s_Aestuariispira_insulae	Met	(0.92)	1.04	0.3806	NA	prevalence	33	5
s_Dialister_succinatiphilus	Met	0.97	1.11	0.3838	NA	prevalence	33	3

s_Bacteroides_caccae	Met	(0.68)	0.81	0.4022	NA	prevalence	33	20
s_Ruminococcus_lactaris	Met	(0.66)	0.79	0.4023	NA	prevalence	33	16
s_Terrisporobacter_mayombei	Met	0.78	0.93	0.4028	NA	prevalence	33	6
s_Neglecta_timonensis	Met	0.68	0.82	0.4029	NA	prevalence	33	21
s_Butyricimonas_faecalis	Met	(1.21)	1.44	0.4036	NA	prevalence	33	2
s_Bacteroides_uniformis	Met	0.26	0.51	0.4048	NA	abundance	33	31
s_Colidextribacter_massiliensis	Met	(0.73)	0.88	0.4110	NA	prevalence	33	13
s_Bacteroides_pectinophilus	Met	(1.22)	1.49	0.4115	NA	prevalence	33	2
s_Raoultibacter_timonensis	Met	(0.80)	0.98	0.4143	NA	prevalence	33	6
s_Lachnospira_eligens	Met	(0.80)	0.58	0.4150	NA	abundance	33	26
s_Akkermansia_muciniphila	Met	(0.65)	0.81	0.4175	NA	prevalence	33	20
s_Prevotella_copri	Met	(4.80)	5.29	0.4178	NA	abundance	33	12
s_Erysipelotrichaceae_bacterium_GAM147	Met	1.01	1.49	0.4179	NA	abundance	33	19
s_Blautia_producta	Met	(1.11)	1.00	0.4206	NA	abundance	33	25
s_Cuneatibacter_caecimuris	Met	(0.87)	1.08	0.4217	NA	prevalence	33	29
s_Hungatella_hathewayi	Met	(0.79)	0.99	0.4251	NA	prevalence	33	26
s_Enterocloster_bolteae	Met	0.39	0.72	0.4251	NA	abundance	33	22
s_Bacteroides_finegoldii	Met	0.82	1.02	0.4252	NA	prevalence	33	4
s_Agathobaculum_butyriciproducens	Met	(1.16)	1.46	0.4264	NA	prevalence	33	31
s_Duodenibacillus_massiliensis	Met	(2.45)	2.53	0.4265	NA	abundance	33	10
s_Sporobacter_termitidis	Met	(1.56)	1.56	0.4276	NA	abundance	33	17
s_Caproiciproducens_galactitolivorans	Met	1.07	1.36	0.4319	NA	prevalence	33	2
s_Dysosmobacter_welbionis	Met	(1.03)	1.33	0.4401	NA	prevalence	33	31
s_Gemmiger_formicilis	Met	0.18	0.45	0.4440	NA	abundance	33	32
s_Bacteroides_rodentium	Met	0.85	1.12	0.4475	NA	prevalence	33	3
s_Parabacteroides_goldsteinii	Met	0.83	1.10	0.4507	NA	prevalence	33	5
s_Coprococcus_catus	Met	(0.71)	0.94	0.4510	NA	prevalence	33	27
s_Lactobacillus_rogosae	Met	(0.66)	0.87	0.4531	NA	prevalence	33	21
s_Cuneatibacter_caecimuris	Met	(0.78)	0.63	0.4584	NA	abundance	33	29
s_Phocaeicola_massiliensis	Met	0.40	0.78	0.4629	NA	abundance	33	14

s_Ruminococcus_bicirculans	Met	0.51	0.97	0.4631	NA	abundance	33	23
s_Sutterella_wadsworthensis	Met	0.32	0.71	0.4724	NA	abundance	33	16
s_Coraliomargarita_akajimensis	Met	(1.04)	1.45	0.4738	NA	prevalence	33	2
s_Dysosmobacter_welbionis	Met	(0.67)	0.50	0.4836	NA	abundance	33	31
s_Alistipes_dispar	Met	(3.04)	2.72	0.4927	NA	abundance	33	6
s_Faecalibacterium_prausnitzii	Met	(0.53)	0.23	0.4930	NA	abundance	33	33
s_Alistipes_putredinis	Met	(0.65)	0.48	0.4944	NA	abundance	33	30
s_Faecalitalea_cylindroides	Met	(0.97)	1.43	0.4964	NA	prevalence	33	2
s_Ruminococcus_sp._JE7A12	Met	(0.61)	0.90	0.4992	NA	prevalence	33	7
s_Erysipelotrichaceae_bacterium_GAM147	Met	(0.52)	0.77	0.5014	NA	prevalence	33	19
s_Bacteroides_intestinalis	Met	0.57	0.85	0.5017	NA	prevalence	33	10
s_Raoultibacter_timonensis	Met	(18.18)	18.24	0.5053	NA	abundance	33	6
s_Anaeromassilibacillus_senegalensis	Met	(1.09)	1.20	0.5073	NA	abundance	33	21
s_Lachnoclostridium_sp._YL32	Met	(0.75)	1.13	0.5084	NA	prevalence	33	3
s_Eubacterium_coprostanoligenes	Met	0.55	1.15	0.5086	NA	abundance	33	24
s_Gemmiger_formicilis	Met	(0.93)	1.43	0.5162	NA	prevalence	33	32
s_Anaeroplasma_varium	Met	(0.67)	1.04	0.5196	NA	prevalence	33	5
s_Phascolarctobacterium_faecium	Met	(1.22)	1.41	0.5210	NA	abundance	33	12
s_Odoribacter_splanchnicus	Met	(0.62)	0.46	0.5212	NA	abundance	33	26
s_Christensenella_massiliensis	Met	0.66	1.32	0.5260	NA	abundance	33	11
s_Bifidobacterium_catenulatum	Met	0.82	1.29	0.5264	NA	prevalence	33	2
s_Bacteroides_stercoris	Met	1.00	1.88	0.5372	NA	abundance	33	11
s_Bacteroides_eggerthii	Met	(0.52)	0.85	0.5412	NA	prevalence	33	10
s_Paraprevotella_clara	Met	1.42	2.43	0.5453	NA	abundance	33	8
s_Bacteroides_cellulosilyticus	Met	(0.47)	0.78	0.5480	NA	prevalence	33	19
s_Blautia_sp._SC05B48	Met	(0.51)	0.29	0.5488	NA	abundance	33	33
s_Christensenella_sp._Marseille.P3954	Met	(0.99)	1.19	0.5521	NA	abundance	33	24
s_Dialister_hominis	Met	(0.69)	1.16	0.5538	NA	prevalence	33	3
s_Dialister_massiliensis	Met	(0.69)	1.16	0.5538	NA	prevalence	33	3
s_Papillibacter_cinnamivorans	Met	(1.39)	1.83	0.5576	NA	abundance	33	12

s_Barnesiella_intestinihominis	Met	(0.55)	0.94	0.5605	NA	prevalence	33	28
s_Collinsella_aerofaciens	Met	(0.60)	1.06	0.5699	NA	prevalence	33	29
s_Dorea_longicatena	Met	(0.63)	0.59	0.5712	NA	abundance	33	32
s_Clostridium_leptum	Met	(3.43)	4.75	0.5724	NA	abundance	33	7
s_Clostridium_saudiense	Met	0.31	0.95	0.5827	NA	abundance	33	18
s_Anaeroplasma_abactoclasticum	Met	(0.57)	1.06	0.5861	NA	prevalence	33	4
s_Phocaeicola_coprocola	Met	0.67	1.27	0.5946	NA	prevalence	33	3
s_Intestinibacter_bartlettii	Met	0.46	0.86	0.5952	NA	prevalence	33	12
s_Eubacterium_xylanophilum	Met	1.73	3.48	0.6004	NA	abundance	33	9
s_Oscillibacter_sp._PEA192	Met	(0.43)	0.82	0.6006	NA	prevalence	33	20
s_Pseudoflavonifractor_phocaeensis	Met	(2.08)	3.14	0.6021	NA	abundance	33	7
s_Vampirovibrio_chlorellavorus	Met	(1.55)	2.39	0.6040	NA	abundance	33	13
s_Oxalobacter_formigenes	Met	0.66	1.28	0.6068	NA	prevalence	33	2
s_Bifidobacterium_faecale	Met	(1.04)	1.49	0.6118	NA	abundance	33	17
s_Clostridium_colinum	Met	(0.50)	0.98	0.6133	NA	prevalence	33	5
s_Neglecta_timonensis	Met	0.14	0.69	0.6137	NA	abundance	33	21
s_Clostridiales_bacterium_CCNA10	Met	0.52	1.02	0.6146	NA	prevalence	33	4
s_Eggerthella_lenta	Met	(0.62)	1.24	0.6147	NA	prevalence	33	3
s_Faecalicatena_fissicatena	Met	0.14	0.71	0.6199	NA	abundance	33	17
s_Pseudoflavonifractor_phocaeensis	Met	(0.45)	0.92	0.6212	NA	prevalence	33	7
s_Catabacter_hongkongensis	Met	0.60	1.63	0.6223	NA	abundance	33	13
s_Faecalicatena_contorta	Met	0.98	2.15	0.6257	NA	abundance	33	7
s_Longicatena_caecimuris	Met	0.46	0.96	0.6287	NA	prevalence	33	7
s_Bacteroides_stercorirosoris	Met	0.59	1.22	0.6313	NA	prevalence	33	3
s_Anaerobutyricum_hallii	Met	(0.03)	0.34	0.6350	NA	abundance	33	33
s_Clostridium_disporicum	Met	0.58	1.24	0.6427	NA	prevalence	33	3
s_Alistipes_finegoldii	Met	(0.79)	1.09	0.6432	NA	abundance	33	17
s_Collinsella_aerofaciens	Met	0.15	0.82	0.6550	NA	abundance	33	29
s_Roseburia_faecis	Met	(0.78)	1.14	0.6586	NA	abundance	33	27
s_Flintibacter_sp._KGMB00164	Met	(0.48)	0.42	0.6632	NA	abundance	33	31

s_Christensenella_timonensis	Met	0.44	1.01	0.6633	NA	prevalence	33	5
s_Phascolarctobacterium_succinatutens	Met	0.39	0.91	0.6669	NA	prevalence	33	7
s_Bifidobacterium_bifidum	Met	0.62	1.43	0.6670	NA	prevalence	33	3
s_Subdoligranulum_variabile	Met	(0.57)	0.66	0.6693	NA	abundance	33	30
s_Faecalimonas_umbilicata	Met	(0.34)	0.80	0.6712	NA	prevalence	33	13
s_Catenibacterium_mitsuokai	Met	(0.63)	1.51	0.6743	NA	prevalence	33	2
s_Agathobacter_ruminis	Met	(0.63)	1.51	0.6778	NA	prevalence	33	2
s_Ruminococcus_callidus	Met	0.40	1.52	0.6801	NA	abundance	33	17
s_Phocaeicola_vulgatus	Met	(0.66)	0.96	0.6834	NA	abundance	33	29
s_Roseburia_faecis	Met	(0.41)	1.01	0.6853	NA	prevalence	33	27
s_Parasutterella_excrementihominis	Met	1.08	3.13	0.6886	NA	abundance	33	10
s_Longibaculum_sp._KGMB06250	Met	(0.41)	1.08	0.7008	NA	prevalence	33	3
s_Turicibacter_sp._H121	Met	(0.40)	1.06	0.7037	NA	prevalence	33	6
s_Sutterella_wadsworthensis	Met	(0.31)	0.84	0.7090	NA	prevalence	33	16
s_Ruminococcus_faecis	Met	(0.49)	1.33	0.7094	NA	prevalence	33	31
s_Alistipes_communis	Met	0.22	1.19	0.7098	NA	abundance	33	17
s_Eubacterium_rectale	Met	(0.38)	1.03	0.7115	NA	prevalence	33	28
s_Papillibacter_cinnamivorans	Met	(0.31)	0.85	0.7124	NA	prevalence	33	12
s_Bacteroides_faecis	Met	(1.37)	2.71	0.7198	NA	abundance	33	7
s_Erysipelothrix_rhusiopathiae	Met	0.34	0.96	0.7200	NA	prevalence	33	7
s_Bifidobacterium_longum	Met	0.12	1.00	0.7221	NA	abundance	33	25
s_Parabacteroides_merdae	Met	(0.67)	1.14	0.7258	NA	abundance	33	21
s_Methanobrevibacter_smithii	Met	(0.39)	1.12	0.7294	NA	prevalence	33	4
s_Longicatena_caecimuris	Met	1.67	4.85	0.7303	NA	abundance	33	7
s_Phocaeicola_massiliensis	Met	0.26	0.77	0.7335	NA	prevalence	33	14
s_Phocaeicola_plebeius	Met	(0.52)	1.53	0.7344	NA	prevalence	33	2
s_Bifidobacterium_longum	Met	(0.29)	0.87	0.7360	NA	prevalence	33	25
s_Ruminococcus_torques	Met	(0.27)	0.81	0.7406	NA	prevalence	33	22
s_Blautia_faecis	Met	(0.40)	1.20	0.7413	NA	prevalence	33	31
s_Roseburia_inulinivorans	Met	0.27	0.83	0.7425	NA	prevalence	33	23

s_Clostridium_spiroforme	Met	0.87	2.99	0.7452	NA	abundance	33	7
s_Pseudoclostridium_thermosuccinogenes	Met	0.31	0.98	0.7516	NA	prevalence	33	5
s_Bacteroides_caccae	Met	(0.73)	1.46	0.7525	NA	abundance	33	20
s_Culturomica_massiliensis	Met	0.38	1.19	0.7533	NA	prevalence	33	2
s_Adlercreutzia_equolifaciens	Met	(0.36)	1.15	0.7566	NA	prevalence	33	3
s_Alistipes_dispar	Met	(0.29)	0.96	0.7619	NA	prevalence	33	6
s_Coprobacter_fastidiosus	Met	(0.50)	0.75	0.7639	NA	abundance	33	11
s_Subdoligranulum_variabile	Met	0.35	1.19	0.7711	NA	prevalence	33	30
s_Senegalimassilia_anaerobia	Met	0.49	2.23	0.7720	NA	abundance	33	7
s_Butyricimonas_virosa	Met	0.34	1.20	0.7782	NA	prevalence	33	3
s_Acholeplasma_axanthum	Met	0.34	1.26	0.7850	NA	prevalence	33	2
s_Candidatus_Liberibacter_americanus	Met	(0.31)	1.13	0.7853	NA	prevalence	33	3
s_Kiloniella_majae	Met	(0.23)	0.91	0.7979	NA	prevalence	33	6
s_Intestinibacillus_massiliensis	Met	(0.27)	1.08	0.8024	NA	prevalence	33	4
s_Azospirillum_brasilense	Met	0.30	1.20	0.8057	NA	prevalence	33	2
s_Caproiciproducens_sp._NJN.50	Met	0.30	1.20	0.8057	NA	prevalence	33	2
s_Holdemania_filiformis	Met	0.30	1.20	0.8057	NA	prevalence	33	2
s_Porphyromonas_gingivalis	Met	0.30	1.20	0.8057	NA	prevalence	33	2
s_Amedibacillus_dolichus	Met	(0.27)	1.09	0.8073	NA	prevalence	33	3
s_Ruminococcus_faecis	Met	(0.15)	0.29	0.8108	NA	abundance	33	31
s_Escherichia_coli	Met	2.77	11.21	0.8129	NA	abundance	33	7
s_Sutterella_massiliensis	Met	(0.26)	1.11	0.8132	NA	prevalence	33	4
s_Roseburia_intestinalis	Met	(0.51)	1.03	0.8137	NA	abundance	33	25
s_Pseudoflavonifractor_capillosus	Met	(0.38)	1.63	0.8170	NA	prevalence	33	2
s_Turicibacter_sp._H121	Met	(2.14)	6.52	0.8203	NA	abundance	33	6
s_Turicibacter_sanguinis	Met	0.23	1.00	0.8206	NA	prevalence	33	4
s_Phocaeicola_dorei	Met	(0.48)	0.96	0.8228	NA	abundance	33	33
s_Blautia_hydrogenotrophica	Met	0.27	1.21	0.8236	NA	prevalence	33	2
s_Dorea_longicatena	Met	0.34	1.55	0.8237	NA	prevalence	33	32
s_Faecalimonas_umbilicata	Met	(0.73)	2.13	0.8272	NA	abundance	33	13

s_Ruminococcus_bromii	Met	(0.22)	1.01	0.8285	NA	prevalence	33	26
s_Bacteroides_eggerthii	Met	(1.06)	3.58	0.8299	NA	abundance	33	10
s_Christensenella_minuta	Met	0.73	4.03	0.8303	NA	abundance	33	7
s_Alistipes_putredinis	Met	0.24	1.13	0.8307	NA	prevalence	33	30
s_Holdemanella_biformis	Met	0.17	0.81	0.8311	NA	prevalence	33	9
s_Eubacterium_ventriosum	Met	0.18	0.85	0.8367	NA	prevalence	33	9
s_Hungateiclostridium_clariflavum	Met	1.11	5.83	0.8376	NA	abundance	33	7
s_Dorea_formicigenerans	Met	(0.18)	0.87	0.8409	NA	prevalence	33	9
s_Blautia_glucerasea	Met	0.01	1.26	0.8425	NA	abundance	33	17
s_Erysipelothrix_rhusiopathiae	Met	0.36	2.70	0.8432	NA	abundance	33	7
s_Bacteroides_stercoris	Met	(0.16)	0.83	0.8459	NA	prevalence	33	11
s_Flavonifractor_plautii	Met	(0.19)	0.98	0.8473	NA	prevalence	33	14
s_Mitsuokella_jalaludinii	Met	0.22	1.17	0.8531	NA	prevalence	33	2
s_Negativibacillus_massiliensis	Met	0.20	1.14	0.8602	NA	prevalence	33	4
s_Ruminococcus_bromii	Met	(0.10)	0.78	0.8642	NA	abundance	33	26
s_Senegalimassilia_anaerobia	Met	(0.15)	0.89	0.8656	NA	prevalence	33	7
s_Oscillibacter_valericigenes	Met	(0.19)	1.15	0.8684	NA	prevalence	33	30
s_Odoribacter_splanchnicus	Met	(0.14)	0.88	0.8713	NA	prevalence	33	26
s_Catabacter_hongkongensis	Met	0.14	0.91	0.8797	NA	prevalence	33	13
s_Christensenella_minuta	Met	(0.14)	0.95	0.8838	NA	prevalence	33	7
s_Bacteroides_uniformis	Met	(0.17)	1.25	0.8910	NA	prevalence	33	31
s_Prevotella_copri	Met	0.11	0.82	0.8921	NA	prevalence	33	12
s_Bifidobacterium_faecale	Met	(0.10)	0.77	0.8951	NA	prevalence	33	17
s_Coprococcus_eutactus	Met	(0.15)	0.70	0.8966	NA	abundance	33	22
s_Eubacterium_siraeum	Met	0.12	0.95	0.8969	NA	prevalence	33	9
s_Bacteroides_thetaiotaomicron	Met	(0.11)	0.87	0.8975	NA	prevalence	33	23
s_Bacteroides_xylanisolvens	Met	(0.11)	1.11	0.9033	NA	abundance	33	17
s_Falcatimonas_natans	Met	(0.12)	1.11	0.9115	NA	prevalence	33	3
s_Terrisporobacter_petrolearius	Met	0.12	1.14	0.9133	NA	prevalence	33	5
s_Duodenibacillus_massiliensis	Met	(0.09)	0.90	0.9171	NA	prevalence	33	10

s_Clostridium_viride	Met	(0.12)	1.14	0.9178	NA	prevalence	33	3
s_Flintibacter_sp._KGMB00164	Met	(0.12)	1.20	0.9179	NA	prevalence	33	31
s_Anaeromassilibacillus_senegalensis	Met	(0.08)	0.78	0.9184	NA	prevalence	33	21
s_Hungatella_xylanolytica	Met	0.10	0.97	0.9201	NA	prevalence	33	4
s_Oscillibacter_sp._PEA192	Met	(0.38)	1.25	0.9216	NA	abundance	33	20
s_Coprococcus_comes	Met	(0.20)	0.35	0.9225	NA	abundance	33	31
s_Bifidobacterium_ruminantium	Met	0.11	1.14	0.9226	NA	prevalence	33	6
s_Faecalicatena_orotica	Met	(0.11)	1.16	0.9229	NA	prevalence	33	2
s_Ruminococcus_sp._JE7A12	Met	(0.65)	3.79	0.9252	NA	abundance	33	7
s_Bacteroides_xylanisolvens	Met	(0.07)	0.79	0.9259	NA	prevalence	33	17
s_Ruthenibacterium_lactatiformans	Met	0.11	1.15	0.9265	NA	prevalence	33	4
s_Enterocloster_bolteae	Met	(0.08)	0.91	0.9298	NA	prevalence	33	22
s_Hespellia_stercorisuis	Met	0.08	0.97	0.9309	NA	prevalence	33	5
s_Eubacterium_siraeum	Met	0.11	4.01	0.9338	NA	abundance	33	9
s_Desulfovibrio_desulfuricans	Met	0.09	1.17	0.9374	NA	prevalence	33	3
s_Eisenbergiella_tayi	Met	(0.39)	1.71	0.9379	NA	abundance	33	15
s_Marseilla_massiliensis	Met	0.08	1.15	0.9452	NA	prevalence	33	2
s_Colidextribacter_massiliensis	Met	(0.33)	1.06	0.9474	NA	abundance	33	13
s_Alistipes_finegoldii	Met	0.05	0.80	0.9505	NA	prevalence	33	17
s_Fusicatenibacter_saccharivorans	Met	(0.29)	0.53	0.9543	NA	abundance	33	33
s_Bacteroides_faecis	Met	0.05	0.88	0.9567	NA	prevalence	33	7
s_Parabacteroides_johnsonii	Met	0.05	0.96	0.9599	NA	prevalence	33	5
s_Christensenella_massiliensis	Met	(0.05)	0.97	0.9617	NA	prevalence	33	11
s_Anaerotignum_faecicola	Met	0.04	0.99	0.9650	NA	prevalence	33	5
s_Tidjanibacter_massiliensis	Met	(0.04)	0.95	0.9672	NA	prevalence	33	6
s_Coprococcus_comes	Met	0.05	1.59	0.9731	NA	prevalence	33	31
s_Clostridium_spiroforme	Met	(0.03)	0.89	0.9749	NA	prevalence	33	7
s_Bacteroides_intestinalis	Met	(0.35)	3.09	0.9759	NA	abundance	33	10
s_Roseburia_inulinivorans	Met	(0.22)	1.16	0.9779	NA	abundance	33	23
s_Paraprevotella_clara	Met	0.02	0.85	0.9788	NA	prevalence	33	8

s_Anaerotignum_lactatifermentans	Met	(0.03)	1.20	0.9823	NA	prevalence	33	2
s_Phascolarctobacterium_succinatutens	Met	(0.38)	6.77	0.9870	NA	abundance	33	7
s_Alistipes_onderdonkii	Met	0.01	0.78	0.9889	NA	prevalence	33	18
s_Flavonifractor_plautii	Met	(0.27)	1.34	0.9900	NA	abundance	33	14
s_Faecalicatena_contorta	Met	(0.01)	0.87	0.9906	NA	prevalence	33	7
s_Blautia_luti	Met	(0.25)	0.35	0.9934	NA	abundance	33	33
s_Akkermansia_muciniphila	Met	(0.25)	1.19	0.9975	NA	abundance	33	20
s_Frisingicoccus_caecimuris	Met	0.00	1.12	0.9990	NA	prevalence	33	3
s_Acholeplasma_axanthum	Met	NA	NA	NA	contrasts can be applied only to factors with 2 or more levels	abundance	33	2
s_Adlercreutzia_equolifaciens	Met	1.46	NA	NA	Fitting error (NA p-value returned from fitting procedure)	abundance	33	3
s_Aestuariispira_insulae	Met	(19.31)	NA	NA	Fitting error (NA p-value returned from fitting procedure)	abundance	33	5
s_Agathobacter_ruminis	Met	NA	NA	NA	contrasts can be applied only to factors with 2 or more levels	abundance	33	2
s_Alistipes_indistinctus	Met	NA	NA	NA	contrasts can be applied only to factors with 2 or more levels	abundance	33	7
s_Amedibacillus_dolichus	Met	0.58	NA	NA	Fitting error (NA p-value returned from fitting procedure)	abundance	33	3
s_Anaeroplasma_abactoclasticum	Met	(4.78)	NA	NA	Fitting error (NA p-value returned from fitting procedure)	abundance	33	4
s_Anaeroplasma_varium	Met	(3.19)	NA	NA	Fitting error (NA p-value returned from fitting procedure)	abundance	33	5

s_Anaerotignum_faecicola	Met	0.27	NA	NA	Fitting error (NA p-value returned from fitting procedure)	abundance	33	5
s_Anaerotignum_lactatifermentans	Met	4.59	NA	NA	Fitting error (NA p-value returned from fitting procedure)	abundance	33	2
s_Anaerotruncus_rubiinfantis	Met	NA	NA	NA	contrasts can be applied only to factors with 2 or more levels	abundance	33	5
s_Azospirillum_brasilense	Met	NA	NA	NA	No data points have the baseline factor level	abundance	33	2
s_Bacteroidales_bacterium_CF	Met	NA	NA	NA	contrasts can be applied only to factors with 2 or more levels	abundance	33	4
s_Bacteroides_clarus	Met	NA	NA	NA	contrasts can be applied only to factors with 2 or more levels	abundance	33	5
s_Bacteroides_finegoldii	Met	(3.59)	NA	NA	Fitting error (NA p-value returned from fitting procedure)	abundance	33	4
s_Bacteroides_koreensis	Met	NA	NA	NA	contrasts can be applied only to factors with 2 or more levels	abundance	33	3
s_Bacteroides_nordii	Met	NA	NA	NA	contrasts can be applied only to factors with 2 or more levels	abundance	33	2
s_Bacteroides_pectinophilus	Met	NA	NA	NA	contrasts can be applied only to factors with 2 or more levels	abundance	33	2
s_Bacteroides_rodentium	Met	0.88	NA	NA	Fitting error (NA p-value returned from fitting procedure)	abundance	33	3
s_Bacteroides_salyersiae	Met	(11.41)	NA	NA	Fitting error (NA p-value returned from fitting procedure)	abundance	33	5

s_Bacteroides_stercorirosoris	Met	(2.86)	NA	NA	Fitting error (NA p-value returned from fitting procedure)	abundance	33	3
s_Bifidobacterium_animalis	Met	0.05	NA	NA	Fitting error (NA p-value returned from fitting procedure)	abundance	33	5
s_Bifidobacterium_bifidum	Met	(3.71)	NA	NA	Fitting error (NA p-value returned from fitting procedure)	abundance	33	3
s_Bifidobacterium_catenulatum	Met	2.95	NA	NA	Fitting error (NA p-value returned from fitting procedure)	abundance	33	2
s_Bifidobacterium_pseudocatenulatum	Met	(1.86)	NA	NA	Fitting error (NA p-value returned from fitting procedure)	abundance	33	3
s_Blautia_hominis	Met	NA	NA	NA	contrasts can be applied only to factors with 2 or more levels	abundance	33	2
s_Blautia_hydrogenotrophica	Met	NA	NA	NA	contrasts can be applied only to factors with 2 or more levels	abundance	33	2
s_Butyricimonas_faecalis	Met	NA	NA	NA	contrasts can be applied only to factors with 2 or more levels	abundance	33	2
s_Butyricimonas_faecihominis	Met	NA	NA	NA	contrasts can be applied only to factors with 2 or more levels	abundance	33	2
s_Butyricimonas_paravirosa	Met	(6.00)	NA	NA	Fitting error (NA p-value returned from fitting procedure)	abundance	33	4
s_Butyricimonas_virosa	Met	NA	NA	NA	contrasts can be applied only to factors with 2 or more levels	abundance	33	3
s_Butyrivibrio_crossotus	Met	NA	NA	NA	contrasts can be applied only to factors with 2 or more levels	abundance	33	5

s_Candidatus_Liberibacter_americanus	Met	NA	NA	NA	contrasts can be applied only to factors with 2 or more levels	abundance	33	3
s_Caproiciproducens_galactitolivorans	Met	NA	NA	NA	contrasts can be applied only to factors with 2 or more levels	abundance	33	2
s_Caproiciproducens_sp._NJN.50	Met	NA	NA	NA	No data points have the baseline factor level	abundance	33	2
s_Catenibacterium_mitsuokai	Met	NA	NA	NA	contrasts can be applied only to factors with 2 or more levels	abundance	33	2
s_Christensenella_timonensis	Met	6.42	NA	NA	Fitting error (NA p-value returned from fitting procedure)	abundance	33	5
s_Clostridiales_bacterium	Met	NA	NA	NA	contrasts can be applied only to factors with 2 or more levels	abundance	33	7
s_Clostridiales_bacterium_CCNA10	Met	(2.50)	NA	NA	Fitting error (NA p-value returned from fitting procedure)	abundance	33	4
s_Clostridium_colinum	Met	31.93	NA	NA	Fitting error (NA p-value returned from fitting procedure)	abundance	33	5
s_Clostridium_disporicum	Met	NA	NA	NA	contrasts can be applied only to factors with 2 or more levels	abundance	33	3
s_Clostridium_sp._BNL1100	Met	(6.22)	NA	NA	Fitting error (NA p-value returned from fitting procedure)	abundance	33	3
s_Clostridium_sporogenes	Met	(14.65)	NA	NA	Fitting error (NA p-value returned from fitting procedure)	abundance	33	5
s_Clostridium_viride	Met	(7.59)	NA	NA	Fitting error (NA p-value returned from fitting procedure)	abundance	33	3

s_Cohaesibacter_haloalkalitolerans	Met	NA	NA	NA	contrasts can be applied only to factors with 2 or more levels	abundance	33	6
s_Coraliomargarita_akajimensis	Met	NA	NA	NA	contrasts can be applied only to factors with 2 or more levels	abundance	33	2
s_Culturomica_massiliensis	Met	(4.82)	NA	NA	Fitting error (NA p-value returned from fitting procedure)	abundance	33	2
s_Desulfovibrio_desulfuricans	Met	NA	NA	NA	contrasts can be applied only to factors with 2 or more levels	abundance	33	3
s_Desulfovibrio_piger	Met	NA	NA	NA	contrasts can be applied only to factors with 2 or more levels	abundance	33	9
s_Dialister_hominis	Met	NA	NA	NA	contrasts can be applied only to factors with 2 or more levels	abundance	33	3
s_Dialister_massiliensis	Met	NA	NA	NA	contrasts can be applied only to factors with 2 or more levels	abundance	33	3
s_Dialister_succinatiphilus	Met	(3.01)	NA	NA	Fitting error (NA p-value returned from fitting procedure)	abundance	33	3
s_Duncaniella_sp._B8	Met	NA	NA	NA	contrasts can be applied only to factors with 2 or more levels	abundance	33	5
s_Eggerthella_lenta	Met	NA	NA	NA	contrasts can be applied only to factors with 2 or more levels	abundance	33	3
s_Enterocloster_clostridioformis	Met	(0.78)	NA	NA	Fitting error (NA p-value returned from fitting procedure)	abundance	33	3
s_Enterococcus_faecium	Met	NA	NA	NA	contrasts can be applied only to factors with 2 or more levels	abundance	33	2
s_Erysipelatoclostridium_ramosum	Met	NA	NA	NA	contrasts can be applied only to factors with 2 or more levels	abundance	33	2

s_Erysipelotrichaceae_bacterium_I46	Met	NA	NA	NA	contrasts can be applied only to factors with 2 or more levels	abundance	33	5
s_Eubacterium_oxidoreducens	Met	NA	NA	NA	contrasts can be applied only to factors with 2 or more levels	abundance	33	4
s_Faecalicatena_orotica	Met	(3.71)	NA	NA	Fitting error (NA p-value returned from fitting procedure)	abundance	33	2
s_Faecalitalea_cylindroides	Met	NA	NA	NA	contrasts can be applied only to factors with 2 or more levels	abundance	33	2
s_Falcatimonas_natans	Met	(4.96)	NA	NA	Fitting error (NA p-value returned from fitting procedure)	abundance	33	3
s_Fournierella_massiliensis	Met	NA	NA	NA	contrasts can be applied only to factors with 2 or more levels	abundance	33	2
s_Frisingicoccus_caecimuris	Met	(8.33)	NA	NA	Fitting error (NA p-value returned from fitting procedure)	abundance	33	3
s_Haemophilus_parainfluenzae	Met	NA	NA	NA	No data points have the baseline factor level	abundance	33	2
s_Hespellia_stercorisuis	Met	9.03	NA	NA	Fitting error (NA p-value returned from fitting procedure)	abundance	33	5
s_Holdemania_filiformis	Met	NA	NA	NA	No data points have the baseline factor level	abundance	33	2
s_Hungateiclostridium_aldrichii	Met	(7.77)	NA	NA	Fitting error (NA p-value returned from fitting procedure)	abundance	33	5
s_Hungatella_xylanolytica	Met	(5.54)	NA	NA	Fitting error (NA p-value returned from fitting procedure)	abundance	33	4

s_Intestinibacillus_massiliensis	Met	(3.40)	NA	NA	Fitting error (NA p-value returned from fitting procedure)	abundance	33	4
s_Lachnoclostridium_sp._YL32	Met	0.77	NA	NA	Fitting error (NA p-value returned from fitting procedure)	abundance	33	3
s_Lachnospira_pectinoschiza	Met	NA	NA	NA	No data points have the baseline factor level	abundance	33	2
s_Lachnospiraceae_bacterium_Choco86	Met	NA	NA	NA	contrasts can be applied only to factors with 2 or more levels	abundance	33	2
s_Longibaculum_sp._KGMB06250	Met	1.55	NA	NA	Fitting error (NA p-value returned from fitting procedure)	abundance	33	3
s_Marseilla_massiliensis	Met	(0.04)	NA	NA	Fitting error (NA p-value returned from fitting procedure)	abundance	33	2
s_Massiliprevotella_massiliensis	Met	NA	NA	NA	contrasts can be applied only to factors with 2 or more levels	abundance	33	4
s_Mediterraneibacter_glycyrrhizinilyticus	Met	NA	NA	NA	contrasts can be applied only to factors with 2 or more levels	abundance	33	2
s_Methanobrevibacter_smithii	Met	NA	NA	NA	contrasts can be applied only to factors with 2 or more levels	abundance	33	4
s_Mitsuokella_jalaludinii	Met	(2.30)	NA	NA	Fitting error (NA p-value returned from fitting procedure)	abundance	33	2
s_Negativibacillus_massiliensis	Met	(2.57)	NA	NA	Fitting error (NA p-value returned from fitting procedure)	abundance	33	4
s_Odoribacter_laneus	Met	NA	NA	NA	contrasts can be applied only to factors with 2 or more levels	abundance	33	2

s_Oxalobacter_formigenes	Met	NA	NA	NA	contrasts can be applied only to factors with 2 or more levels	abundance	33	2
s_Parabacteroides_goldsteinii	Met	NA	NA	NA	contrasts can be applied only to factors with 2 or more levels	abundance	33	5
s_Parabacteroides_johnsonii	Met	(1.99)	NA	NA	Fitting error (NA p-value returned from fitting procedure)	abundance	33	5
s_Phocaeicola_coprocola	Met	NA	NA	NA	contrasts can be applied only to factors with 2 or more levels	abundance	33	3
s_Phocaeicola_plebeius	Met	NA	NA	NA	contrasts can be applied only to factors with 2 or more levels	abundance	33	2
s_Phocea_massiliensis	Met	NA	NA	NA	contrasts can be applied only to factors with 2 or more levels	abundance	33	2
s_Porphyromonas_gingivalis	Met	NA	NA	NA	No data points have the baseline factor level	abundance	33	2
s_Prevotella_loescheii	Met	NA	NA	NA	contrasts can be applied only to factors with 2 or more levels	abundance	33	2
s_Prevotella_shahii	Met	8.67	NA	NA	Fitting error (NA p-value returned from fitting procedure)	abundance	33	3
s_Pseudoclostridium_thermosuccinogenes	Met	(11.95)	NA	NA	Fitting error (NA p-value returned from fitting procedure)	abundance	33	5
s_Pseudoflavonifractor_capillosus	Met	NA	NA	NA	contrasts can be applied only to factors with 2 or more levels	abundance	33	2
s_Ruminiclostridium_josui	Met	1.53	NA	NA	Fitting error (NA p-value returned from fitting procedure)	abundance	33	4
s_Ruthenibacterium_lactatiformans	Met	NA	NA	NA	contrasts can be applied only to factors with 2 or more levels	abundance	33	4

s_Salmonella_enterica	Met	NA	NA	NA	contrasts can be applied only to factors with 2 or more levels	abundance	33	2
s_Streptococcus_thermophilus	Met	0.21	NA	NA	Fitting error (NA p-value returned from fitting procedure)	abundance	33	4
s_Sutterella_faecalis	Met	NA	NA	NA	contrasts can be applied only to factors with 2 or more levels	abundance	33	2
s_Sutterella_massiliensis	Met	NA	NA	NA	contrasts can be applied only to factors with 2 or more levels	abundance	33	4
s_Tepidibaculum_saccharolyticum	Met	NA	NA	NA	contrasts can be applied only to factors with 2 or more levels	abundance	33	3
s_Terrisporobacter_petrolearius	Met	(1.60)	NA	NA	Fitting error (NA p-value returned from fitting procedure)	abundance	33	5
s_Turicibacter_sanguinis	Met	(3.78)	NA	NA	Fitting error (NA p-value returned from fitting procedure)	abundance	33	4
s_Victivallis_vadensis	Met	NA	NA	NA	contrasts can be applied only to factors with 2 or more levels	abundance	33	4
s_Anaerobutyricum_hallii	Met	NA	NA	NA	All logistic values are the same	prevalence	33	33
s_Anaerostipes_hadrus	Met	NA	NA	NA	All logistic values are the same	prevalence	33	33
s_Blautia_luti	Met	NA	NA	NA	All logistic values are the same	prevalence	33	33
s_Blautia_sp._SC05B48	Met	NA	NA	NA	All logistic values are the same	prevalence	33	33
s_Blautia_wexlerae	Met	NA	NA	NA	All logistic values are the same	prevalence	33	33
s_Faecalibacterium_prausnitzii	Met	NA	NA	NA	All logistic values are the same	prevalence	33	33
s_Fusicatenibacter_saccharivorans	Met	NA	NA	NA	All logistic values are the same	prevalence	33	33
s_Phocaeicola_dorei	Met	NA	NA	NA	All logistic values are the same	prevalence	33	33
